# On the Anatomy of Medical Progress Within an Overlapping Generations Economy

**DOI:** 10.1007/s10645-020-09360-3

**Published:** 2020-04-06

**Authors:** Ivan Frankovic, Michael Kuhn, Stefan Wrzaczek

**Affiliations:** Wittgenstein Centre (Univ. Vienna, IIASA, VID/ÖAW), Vienna Institute of Demography, Vienna, Austria

**Keywords:** Life-cycle model, Longevity, Health care, Medical innovation, Overlapping generations, Value of life, D15, I11, I12, J11, J17, O31, O41

## Abstract

We study medical progress within a two-sector economy of overlapping generations subject to endogenous mortality. Individuals demand health care with a view to lowering mortality over their life-cycle. We characterise the individual optimum and the general equilibrium, and study the impact of a major medical innovation leading to an improvement in the effectiveness of health care. We find that general equilibrium effects dampen strongly the increase in health care usage following medical innovation. Moreover, an increase in savings offsets the negative impact on GDP per capita of a decline in the support ratio. Finally, we show that the reallocation of resources between the final goods and health care sector, following the innovation, plays a crucial role in shaping the general equilibrium impact.

## Introduction

A consensus has emerged that medical progress is driving both the increase in health care spending and the increase in longevity (e.g. Cutler 2004; Chandra and Skinner 2012; Chernew and Newhouse 2012).^1^ Recent analysis by Fonseca et al. (2013) shows that about 30% of health care spending growth in the US over the period 1965–2005 can be explained by medical progress, with improved health insurance coverage explaining 6% and income growth explaining 4%.^2^$$^{,}$$^3^ At the same time, medical progress explains most of the increase in life expectancy over the period of observation, which in welfare terms more than offsets the greater spending. These findings echo, at aggregate level, earlier results by Cutler and Huckman (2003) and Cutler (2007) who find that the technological improvements in the treatment of heart disease over the 1980s and 1990s were generating benefits from increased survival, the value of which was more than compensating the boost to health care costs.^4^

The current line of inquiry remains to a large extent silent about the general equilibrium effects of medical progress. Indeed, there is strong evidence that medical innovations tend to boost the utilisation of health care (e.g. Baker et al. 2003; Cutler and Huckman 2003; Wong et al. 2012; Roham et al. 2014). Given that the main concern about the expanding health share in the economy lies with its absorption of resources that may be employed more productively in other sectors of the economy (Pauly and Saxena 2012; Kuhn and Prettner 2016) it is then surprising that the role of medical progress in this has not yet received more attention. An examination of this concern warrants a general equilibrium analysis that keeps track of the way in which medical progress drives sectoral change in the economy and of the way in which the induced price responses feed back into the pattern of demand.

While a number of recent articles have investigated the role of medical progress in various settings (for a detailed discussion see below), these works abstract from pathways of medical progress that are crucial for understanding the macroeconomic impact or remain intransparent about the underlying transmission mechanisms at both the individual and the macroeconomic level. In this paper, we seek to open the “black box” and study in detail these channels analytically and numerically. So doing enables us to identify a number of features relating to the economic impact of medical innovations that are crucial from both a modelling and a policy perspective.

Specifically, we examine the impact of a medical innovation on individual life-cycle outcomes and on economic performance by analysing an OLG model in which individuals demand health care in order to increase longevity. Health care is supplied within a medical sector, competing for capital and labour with a final goods production sector. We characterise the optimal life-cycle allocation in terms of consumption and health care and show how it evolves with age, depending on the various prices and on the state of medical technology. We characterise the value of life, i.e. the monetary value individuals attach to their survival, as a key determinant of the demand for health care. The value of life will prove to be an important link between macroeconomic changes and their impact on individual choices. Solving the profit maximisation problem of perfectly competitive providers within the final goods and health care sectors, we can characterise the optimal structure of supply and factor demand as well as the aggregate dynamics. Medical progress is modelled as an increase in the effectiveness of health care in lowering mortality. It thus has the character of a product innovation rather than a process innovation, which would entail an increase in total factor productivity in the health care sector.^5^

We employ our model to analyse numerically the impact of medical progress on the provision of health care. Based on a steady-state benchmark scenario that is calibrated to represent the US economy in the year 2003, we study the impact of a stylised and major medical innovation that raises life expectancy by a little more than one year, this being broadly consistent with the increase in life expectancy brought about by the US cardiac revolution during the 80s and 90s (Cutler 2007). We adopt such a quasi-experimental approach to trace out the micro- and macroeconomic adjustment processes that take the economy into a new steady state. The deliberate abstraction from interfering macroeconomic time trends, such as conventional productivity growth, allows a clean analytical and numerical identification of the impact of medical innovation.

Our key results include the following. At the individual level, we find that while medical innovation boosts the demand for health care in partial equilibrium, this effect is more than halved once price responses are accounted for. This illustrates the relevance of general equilibrium effects in OLG settings, similar to a finding by Heijdra and Mierau (2012) in the context of annuity moral hazard. At the macroeconomic level, we find that medical progress tends to increase health care spending predominantly through a boost in utilisation. Although this leads to a sizeable increase in the health expenditure share in GDP, reflecting the sectoral reallocation from final goods production to health care, the overall level of GDP per capita remains unaffected. This is because the drop in the employment rate that comes with a disproportionate increase in survival amongst the retired population is neutralised by an increase in labour productivity that is brought about by capital deepening, similar to the unbalanced growth mechanism in Acemoglu and Guerrieri (2008). In our model, the accumulation of additional capital is induced by the increase in longevity and the prospect for individuals to purchase more effective health care in their old age.^6^ Moreover, our model is able to explain medical price inflation as a general equilibrium outcome.^7^ Indeed, medical price inflation is consistent with our assumption that medical progress predominantly comes in the form of product innovations (i.e. more effective medical care) rather than in the form of process innovation (i.e. more efficient production of health care) which should have led to a decline in medical prices. Finally, we find that survival enhancing medical innovations tend to depress the value of survival over large parts of the life-course. On the one hand, this reflects a reduction in consumption levels; on the other hand, it implies that the price of medical care *per life-year gained *has fallen in spite of an increase in the nominal price of health care, a result that is in line with empirical evidence (Cutler et al. 1998; Lucarelli and Nicholson 2009; Dunn 2012; Lakdawalla et al. 2015; Hult et al. 2018).

Our work ties in with an emergent literature on the macroeconomic impact of medical progress. Similar to our approach, Suen (2009) considers the impact of life-saving health care, the productivity of which is raised by medical change. While his quantitative findings about the increase in life expectancy and health care expenditure are plausible, his modelling differs in important respects: Suen (2009) considers a single sector economy with health care spending being deducted from consumption. Partly for this reason, he does not model an endogenous price for health care but rather imposes an exogenous price trajectory. Medical progress, understood as productivity growth in the health care sector, is captured by a * declining* price of medical care, boosting the demand for it. However, both the fall in the price for medical care and the assertion of sizeable productivity growth in the health care sector run counter to the evidence compiled in footnote 5. In contrast, our two sector setting with medical progress modelled as product innovation is fully consistent with the price trends. Fonseca et al. (2013) simulate a rich life-cycle model and provide quantitatively plausible trends for the impact of medical progress and income growth on health care expenditure. However, as they consider a partial equilibrium setting, their model remains uninformative about the macroeconomic pathways of medical progress. Kelly (2017) studies the response of a neoclassical economy with a medical sector to changes in total factor productivity and in the productivity of health care. In contrast to our approach, the health care sector modelled in Kelly (2017) is not employing domestic production factors. With medical progress thus being unrelated to factor prices and final goods production, no insights can be gained on the sectoral dynamics. Koijen et al. (2016) study the interaction between financial and real health care markets and find that the premium associated with regulatory risk for e.g. pharmaceutical companies lowers research and development (R&D) investments and thereby contains growth of health care expenditure. Schneider and Winkler (2016) study an endogenous growth economy in which overlapping cohorts of individuals invest in health care in order to lower mortality. Comparing the balanced growth paths associated with different states of medical technology, they find that the technology leading to a higher life expectancy imposes a drag on economic growth but leads to a welfare gain. By its more realistic modelling of the individual life-cycle the present work differs from Koijen et al. (2016) and Kelly (2017) who consider an infinitely lived representative individual, as well as from Schneider and Winkler (2016) who consider a Blanchard-Yaari model with endogenous but age-unspecific mortality and perfect annuitisation. Realistic demographic modelling is important in as far as the economic impact of medical progress hinges on the way it shifts the age distribution of the population.^8^$$^{,}$$^9^ Studying (1) the role of health insurance expansion as a driver of medical progress and (2) medical progress as a driver of the longevity gap in the US, Frankovic and Kuhn (2018, 2019) consider a model that is similar to the one presented here, but calibrate it to reflect the dynamics of longevity and health care expenditure growth.^10^ This requires the additional consideration of productivity growth, health insurance expansion and social security expansion as competing drivers of health care spending, which in turn obfuscates the analysis of the transmission of medical progress per se. By isolating the mechanics of medical progress as a crucial driver in itself, the present work contributes an important backdrop to the more quantitative modelling.

Overall, the modelling in Frankovic and Kuhn (2018, 2019) shows that a full general equilibrium analysis is warranted in particular to capture (1) an increase in the price of health care, as driven by Baumol (1967)-style effects that arise from productivity growth in the production sector but also by medical progress, which in turn much dampens the demand increase for health care; (2) the macroeconomic impacts of the increase in the economy-wide capital intensity that is driven by a savings response to greater longevity and enhanced medical treatment options in old age; and (3) the offsetting impact of an increase in old-age dependency if medical progress allows for lives to be saved predominantly after retirement. While the findings in Frankovic and Kuhn (2018, 2019) are fully in line with the channels identified in the present paper, they also reveal the importance of two additional channels that have been (deliberately) shut down in the present paper: First, the concomitant presence of productivity growth, as induced by conventional technological progress, complements medical progress in the demand for health care in as far as it defuses the trade-off with consumption. By boosting the value of survival income and consumption growth induce an ongoing increase in the demand for health care and, implicitly, in the demand for medical innovations. Second, Frankovic and Kuhn (2019) show that medical progress tends to increase the socio-economic gradient in longevity not the least because of the complementarity between income and medical progress as drivers of the demand for health care.

The remainder of the paper is structured as follows: The following section is devoted to a presentation of the model; Sects. 3 and 4 characterise the individual life-cycle allocation and the general equilibrium of the economy, respectively; Sect. 5 provides an analytical assessment of the impact of medical progress; Sect. 6 presents the numerical analysis before Sect. 7 wraps up. Some of the proofs have been relegated to an “Appendix”.

## The Model

We consider an OLG economy in which individuals choose consumption and health care over their life-course. Individuals are assumed to be representative within each cohort and are indexed by their age *a* at time *t*, with $$ t_{0}=t-a$$ denoting the birth year of an individual aged *a* at time *t*. At each age, the representative individual is subject to a mortality risk, where $$S(a,t)=\exp \left[ -\int \nolimits _{0}^{a}\mu ({\widehat{a}},h(\widehat{ a}, t-a+{\widehat{a}}),M\, (t-a+{\widehat{a}}))d{\widehat{a}}\right] $$ is the survival function at (*a*, *t*),  with $$\mu (a,h(a,t),M(t))$$ denoting the force of mortality. Following Kuhn et al. (2010, 2011, 2015) we assume that mortality can be lowered by the consumption of a quantity *h*(*a*, *t*) of health care. In addition, we assume that mortality depends on the state of the medical technology *M*(*t*) at time *t*. More specifically, we assume that the mortality rate $$\mu (a,h(a,t),M(t))$$ satisfies

$$\begin{aligned} \mu (a,h(a,t),M(t)) &\in \left( 0,{\tilde{\mu }}(a,t)\right] \quad \forall \left( a,t\right) ; \\ \mu _{h}(\cdot ) &< 0,\ \mu _{hh}(\cdot )>0; \\ \mu _{h}(a,0,M(t)) &= -\infty ,\ \mu _{h}(a,\infty ,M(t))=0\quad \forall \left( a,t\right) ; \end{aligned}$$

where ${\tilde{\mu }}(a,t)=\mu (a,0,M(t))$ is the “ natural ” mortality rate for an individual aged *a* at time *t* when no health care is consumed. By purchasing health care, the representative individual can lower the instantaneous mortality rate, and can thereby improve survival prospects, but can only do so with diminishing returns.^11^

In regard to medical technology, we assume the following properties

$$\begin{aligned} \mu _{M}(\cdot )<0,\ \mu _{MM}(\cdot )\ge 0,\ \mu _{hM}(\cdot )\gtreqqless 0\quad \forall \left( a,t\right) . \end{aligned}$$

Hence, medical technology contributes towards reductions in mortality ($\mu _{M}(\cdot )<0$) with (weakly) decreasing returns. We leave it open, however, whether for any given positive level of health care, $h(a,t)>0$, medical technology is complementing the consumption of health care ($\mu _{hM}(a,h(a,t),M(t))\le 0$) or substituting it ($\mu _{hM}(a,h(a,t),M(t))>0$).

Individuals enjoy period utility $u(c(a,t)-c_{0})$ from consumption *c*(*a*, *t*),  where $c_{0}\ge 0$ denotes a level of subsistence consumption. Period utility is increasing and concave: $u_{c}(\cdot )>0$, $u_{cc}(\cdot )\le 0$. In addition, we assume the Inada condition $u_{c}(0)=+\infty$. Individuals maximise the present value of their expected life-cycle utility

1 $$\begin{aligned} \max _{c(a,t),h(a,t)}\int _{0}^{\omega }e^{-\rho a}u(c(a,t)-c_{0})S(a,t)da \end{aligned}$$

by choosing a stream of consumption and health care on the interval $\left[ 0,\omega \right] ,$ with $\omega$ denoting the maximal possible age, with $\rho \ge 0$ denoting the rate of time preference, and with *S*(*a*, *t*),  defined above, denoting the survival function.

The individual faces as constraints the dynamics of survival *S*(*a*, *t*) and individual assets *k*(*a*, *t*).^12^ The survival dynamics are described by

2 $$\begin{aligned} \overset{\cdot }{S}(a,t)=-\mu (a,h(a,t),M(t))S(a,t). \end{aligned}$$

Here, (2) describes the reduction of survival according to the force of mortality. While for the sake of simplification we are subsequently referring to $S\left( a,t\right)$ as survival, the function may be interpreted as a more general measure of health that is subject to depreciation over the life-course (Chandra and Skinner 2012, Kuhn et al. 2015). Indeed, (2) not only describes the mortality process, but also proxies for the gradual decline in health over the life-course, as is documented by the gradual accumulation of health deficits (e.g. Rockwood and Mitnitski 2007; Abeliansky and Strulik 2018). With our focus being on an individual representing a whole cohort, it is plausible to assume that the consumption of health care slows down the decline in health but cannot reverse it.^13^ Furthermore, assuming that utility from consumption and utility from good health are multiplicatively separable, one can easily generalise the interpretation of (1) to include not only health-dependent duration of life but also health-dependent quality of life. We assume that the survival function is bounded between 1 at birth and 0 at the maximum feasible age $\omega$, implying the boundary conditions

3 $$\begin{aligned} S(0,t_{0})=1,\quad S(\omega ,t_{0}+\omega )=0. \end{aligned}$$

The asset dynamics are described by

4 $$\begin{aligned}{\dot{k}}(a,t) &=r\left( t\right) k(a,t)+l(a)w(t)-c(a,t) \nonumber \\&\quad -\phi \left( a,t\right) p_{H}(t)h(a,t)-\tau \left( a,t\right) +\pi \left( a,t\right) +s(t). \end{aligned}$$

According to (4) an individual’s stock of assets *k*(*a*, *t*) (1) increases with the return on the current stock, where $r\left( t\right)$ denotes the interest rate at time *t*; (2) increases with earnings *l*(*a*)*w*(*t*),  where *w*(*t*) denotes the wage rate at time *t*, and where *l*(*a*) denotes an individual’s age-dependent effective labour supply; (3) decreases with consumption, the price of consumption goods being normalised to one; (4) decreases with private health expenditure, $\phi \left( a,t\right) p_{H}(t)h(a,t),$ where $p_{H}(t)$ denotes the price for health care, and where $\phi \left( a,t\right)$ denotes an (*a*, *t*)-specific rate of coinsurance; (5) decreases with an (*a*, *t*)-specific tax, $\tau \left( a,t\right) ;$ (6) increases with (*a*, *t*)-specific benefits $\pi \left( a,t\right) ;$ and (7) increases with a transfer *s*(*t*) by which the government redistributes accidental bequests in a lump-sum fashion. Here, we follow Suen (2009), Ludwig et al. (2012) and Zhao (2014) by considering a setting without an annuity market. We assume that individuals enter and leave the life-cycle without assets, implying the boundary conditions

5 $$\begin{aligned} k(0,t_{0})=k(\omega ,t_{0}+\omega )=0. \end{aligned}$$

While the setting without annuity market is well in line with evidence that few individuals annuitise their wealth (e.g. Warshawsky 1988; Reichling and Smetters 2015), we have also considered a specification with imperfect annuities yielding a return $r\left( t\right) +\theta {\overline{\mu }}\left( a,t\right) ,$ where $\theta \in \left[ 0,1\right]$ and where ${\overline{\mu }}\left( a,t\right) =\mu (a,h^{*}(a,t),M(t))$ is the expected mortality, given the equilibrium level of health care $h^{*}(a,t).$ Following Heijdra and Mierau (2012) in considering a scenario with $\theta =0.7$, we obtain qualitatively similar results to those reported in this paper.

Denoting by $B(t-a)$ the size of the birth cohort at $t_{0}=t-a$, the cohort aged *a* at time *t* has the size

$$\begin{aligned} N(a,t)=S(a,t)B(t-a). \end{aligned}$$

By aggregating over the age-groups who are alive at time *t* we obtain the following expressions for the population size,^14^ aggregate capital stock, aggregate effective labour supply, aggregate consumption, and aggregate demand for health care, each at time *t*:

6 $$\begin{aligned} N(t) &= \int _{0}^{\omega }N(a,t)da, \nonumber \\ K(t) &= \int _{0}^{\omega }k(a,t)N(a,t)da, \nonumber \\ L(t) &= \int _{0}^{\omega }l\left( a\right) N(a,t)da, \nonumber \\ C(t) &= \int _{0}^{\omega }c(a,t)N(a,t)da, \end{aligned}$$

7 $$\begin{aligned} H(t) &= \int _{0}^{\omega }h(a,t)N(a,t)da. \end{aligned}$$

The economy consists of a manufacturing sector and a health care sector. In the manufacturing sector a final good is produced by employment of capital $$ K_{Y}(t)$$ and labour $$L_{Y}(t)$$ according to a neoclassical production function $$Y(K_{Y}(t),A\left( t\right) L_{Y}(t)),$$ with $$A\left( t\right) $$ measuring the state of labour augmenting technology. A manufacturer’s profit can then be written as

8 $$\begin{aligned} V_{Y}(t)=Y(K_{Y}(t),A\left( t\right) L_{Y}(t))-w(t)L_{Y}(t)-\left[ \delta +r\left( t\right) \right] K_{Y}(t), \end{aligned}$$

where $\delta$ denotes the depreciation rate of capital.

Health care goods and services are produced by employment of labour $$ L_{H}(t),$$ and capital $$K_{H}(t)$$ according to the neoclassical production function $$F(K_{H}(t),L_{H}(t)).$$ Recalling the price for health care $$ p_{H}\left( t\right) ,$$ the profit of a health care provider is then given by

9 $$\begin{aligned} V_{H}(t) =p_{H}\left( t\right) F(K_{H}(t),L_{H}(t))-w(t)L_{H}(t) -\left[ \delta +r\left( t\right) \right] K_{H}(t), \end{aligned}$$

where we assume that capital depreciates at the same rate across both sectors. Note that the presence of perfect competition together with a neoclassical production function in the two sectors implies $$ V_{Y}(t)=V_{H}(t)=0$$ in equilibrium.

The government and/or a third-party payer (e.g. a health insurer) raise taxes (or contribution rates, e.g. insurance premiums) for the purpose of co-financing health care at the rate $1-\phi \left( a,t\right)$ and of paying out transfer payments $\pi \left( a,t\right)$. More specifically, we let $\pi \left( a,t\right)$ refer to pension benefits, implying that

$$\begin{aligned} \pi \left( a,t\right) =\left\{ \begin{array}{l} 0\Leftrightarrow a<a_{R} \\ \pi \ge 0\Leftrightarrow a\ge a_{R}, \end{array} \right. \end{aligned}$$

with $\pi$ a uniform pension benefit and $a_{R}$ the retirement age. In such a setting we also have

$$\begin{aligned} l\left( a\right) =\left\{ \begin{array}{l} {\widehat{l}}\left( a\right) \ge 0\Leftrightarrow a<a_{R} \\ 0\Leftrightarrow a\ge a_{R}, \end{array} \right. \end{aligned}$$

implying that individuals supply a certain age-specific amount of labour ${\widehat{l}}\left( a\right)$ up to their (mandatory) retirement at age $a_{R}$, from which point onwards earnings, $w(t){\widehat{l}}\left( a\right)$, are replaced by retirement benefits, $\pi$.

Likewise, $\tau \left( a,t\right)$ are age-specific taxes. We could distinguish these into taxes used to finance health care payments (or health insurance premiums), $\tau _{H}\left( a,t\right) ,$ and social security contributions, $\tau _{\Pi }\left( a,t\right) ,$ where $\tau \left( a,t\right) =\tau _{H}\left( a,t\right) +\tau _{\Pi }\left( a,t\right) .$ Furthermore, we could, in principle distinguish between lump-sum and labour income taxes, $\tau _{j}\left( a,t\right) ={\widehat{\tau }}_{j}\left( a,t\right) l\left( a\right) w(t)$, with $j=H,\Pi .$ As long as we assume a unified government budget and an exogenous labour supply, it is sufficient to consider $\tau \left( a,t\right) .$

Assuming that the government budget must be balanced within each period *t* we obtain the constraint

10 $$\begin{aligned}&\int _{0}^{\omega }\left\{ \left[ 1-\phi \left( a,t\right) \right] p_{H}\left( t\right) h(a,t) +\pi \left( a,t\right) \right. \nonumber \\&\qquad \left. -\tau \left( a,t\right) \right\} S(a,t)B(t-a)da=0. \end{aligned}$$

Finally, we assume that total accidental bequests are redistributed in a lump-sum way according to^15^

11 $$\begin{aligned} s(t)=\frac{1}{N(t)}\int _{0}^{\omega }\mu (a,t)k(a,t)N(a,t)da. \end{aligned}$$

## Life-Cycle Optimum

In “Appendix 1” we show that the solution to the individual life-cycle problem is described by the following two sets of conditions^16^

12 $$\begin{aligned}&\frac{u_{c}\left( a,t \right) }{\exp \left\{ -\int _{a}^{ {\widehat{a}}}\left[ \rho +\mu \left( \widehat{{\widehat{a}}},t+\widehat{ {\widehat{a}}}-a\right) \right] d\widehat{{\widehat{a}}}\right\} u_{c}\left( {\widehat{a}},t+{\widehat{a}}-a \right) } \nonumber \\&\quad =\exp \left[ \int _{a}^{{\widehat{a}}}r\left( t+\widehat{{\widehat{a}}} -a\right) d\widehat{{\widehat{a}}}\right] , \end{aligned}$$

13 $$\begin{aligned}&-\mu _{h}\left( a,t\right) \psi \left( a,t\right) =\phi \left( a,t\right) p_{H}\left( t\right) \quad \forall \left( a,t\right) , \end{aligned}$$

describing the optimal pattern of consumption $c\left( a,t\right)$ and the demand for health care $h\left( a,t\right)$, respectively, of an individual aged *a* at time *t*. Condition (12) is the well-known Euler equation, requiring that the marginal rate of intertemporal substitution between consumption at any two ages/years $\left( a,t\right)$ and $\left( {\widehat{a}},t+{\widehat{a}}-a\right)$ equals the compound interest.^17^ In the absence of annuity markets, the uninsured mortality risk can be interpreted as an additional factor of discounting, implying an effective discount rate $\rho +\mu \left( a,t\right)$ at any $\left( a,t\right)$.

Condition (13) requires that at each $\left( a,t\right)$ the marginal value of health care, $-\mu _{h}\left( a,t\right) \psi \left( a,t\right) ,$ equals its effective price, $\phi \left( a,t\right) p_{H}\left( t\right) .$ The marginal value of health care is given by the marginal effect of health care on mortality, $-\mu _{h}\left( a,t\right)$, weighted with the private value of life (VOL). The private VOL is defined by

14 $$\begin{aligned} \psi \left( a,t\right) :=\int _{a}^{\omega } v\left( {\widehat{a}},t+{\widehat{a}} -a\right) R\left( {\widehat{a}},a\right) d{\widehat{a}}, \end{aligned}$$

with

15 $$\begin{aligned} v\left( a,t \right) := \frac{u\left( a,t\right) }{u_{c}\left( a,t\right) } , \end{aligned}$$

and

16 $$\begin{aligned} R\left( {\widehat{a}},a\right) :=\exp \left[ -\int _{a}^{{\widehat{a}}}r\left( t+ \widehat{{\widehat{a}}}-a\right) d\widehat{{\widehat{a}}}\right] , \end{aligned}$$

and amounts to the discounted stream of annual consumer surplus, $v\left( {\widehat{a}},t+{\widehat{a}} -a\right)$ taken over the expected remaining life-course $[ a,\omega ].$^18^ It measures an individual’s willingness to pay for surviving through (*a*, *t*).

For a given set of prices, the evolution of consumption with age is described by (for a derivation see “Appendix 1”)

17 $$\begin{aligned} \overset{\cdot }{c}=\frac{u_{c}}{u_{cc}}\left( \rho -r+\mu \right) . \end{aligned}$$

Noting that $u_{cc}<0,$ it is readily seen that consumption tends to increase over the life-cycle if and only if $r-\rho >\mu .$ In the absence of an annuity market, the uninsured mortality risk imposes a downward drag on consumption over the life-cycle and implies that consumption will eventually decrease with age when mortality $\mu$ grows sufficiently high.

For a given set of prices and a given state of the medical technology, the demand for health care evolves with age as described by (for a derivation see “Appendix 1”)

18 $$\begin{aligned} \overset{\cdot }{h}=\frac{-1}{\mu _{hh}}\left[ \mu _{ha}+\mu _{h}\left( \frac{\overset{\cdot }{\psi }}{\psi }-\frac{\overset{\cdot }{\phi }}{\phi } \right) \right] . \end{aligned}$$

Noting that $\mu _{hh}>0,$ the impact of age on the consumption of health care involves three forces: (1) the changing effectiveness of health care with age $\mu _{ha}$, a stronger (weaker) effectiveness with age, $\mu _{ha}<0$ ($>0$), implying an increase (decrease) in health care;  (2) the rate at which the VOL changes with age, a decrease implying a reduction in health care; and (3) changes with age in the co-insurance rate, $\phi$, as e.g. during a transition from private to public health insurance at the onset of retirement.

Differentiating (14) with respect to age, we obtain the dynamics of the private VOL as

19 $$\begin{aligned} \overset{\cdot }{\psi }\left( a,t\right) =r\left( t\right) \psi \left( a,t\right) - v\left( a,t \right) . \end{aligned}$$

Thus, the private VOL increases with the interest rate and declines over time as the consumer surplus from a succession of life-years lived is written off.

## General Equilibrium

Perfectly competitive firms in the production sector choose labour $L_{Y}(t)$ and capital $K_{Y}\left( t\right)$ so as to maximise period profit (8). The first-order conditions imply

20 $$\begin{aligned} r\left( t\right) &= Y_{K_{Y}}\left( t\right) -\delta \end{aligned}$$

21 $$\begin{aligned} w\left( t\right) &= Y_{L_{Y}}\left( t\right) , \end{aligned}$$

i.e. the factor prices are equalised with their respective marginal products.

Likewise, perfectly competitive providers of health care choose labour $$ L_{H}(t)$$ and capital $$K_{H}\left( t\right) $$ so as to maximise period profit (9). From the first-order condition we obtain

22 $$\begin{aligned} r\left( t\right) &= p_{H}\left( t\right) F_{K_{H}}\left( t\right) -\delta \end{aligned}$$

23 $$\begin{aligned} w\left( t\right) &= p_{H}\left( t\right) F_{L_{H}}\left( t\right) . \end{aligned}$$

Combining these conditions with (20) and (21) we obtain

24 $$\begin{aligned} p_{H}\left( t\right) =\frac{Y_{L_{Y}}\left( t\right) }{F_{L_{H}}\left( t\right) }=\frac{Y_{K_{Y}}\left( t\right) }{F_{K_{H}}\left( t\right) }, \end{aligned}$$

implying that capital and labour inputs are distributed across the production and health care sector in a way that equalises the marginal rate of transformation (i.e. the relative output gain in production as compared to the output loss in health care from re-allocating one factor unit from health care into production) with the price for health care. The higher the latter, the greater the marginal rate of transformation, implying that more workers will be allocated to the health care sector. With appropriate Inada conditions, $Y_{L_{Y}}(K_{Y},0)=Y_{K_Y}\,(0,AL_{Y})=\infty$ and $F_{L_{H}}(K,0)=F_{K_H}(0,L_{H})=\infty$ we always have an interior allocation with $L_{H}(t)=L(t)-L_{Y}(t)\in \left( 0,L\left( t\right) \right)$ and $K_{H}\left( t\right) =K\left( t\right) -K_{Y}\left( t\right) \in \left( 0,K\left( t\right) \right) .$

Our setting involves four markets: two input markets for capital and labour, respectively; and two output markets for health care and for final goods, respectively. From the four market clearing conditions

$$\begin{aligned} K_{Y}(t)+K_{H}(t) &= K(t), \\ L_{Y}(t)+L_{H}(t) &= L(t) \\ F(t) &= H(t), \\ Y(t) &= C\left( t\right) +\overset{\cdot }{K}\left( t\right) +\delta K(t), \end{aligned}$$

we obtain a set of equilibrium prices $\left\{ r^{*}\left( t\right) ,w^{*}\left( t\right) ,p_{H}^{*}\left( t\right) \right\}$ as well as the level of net capital accumulation $\overset{\cdot }{K}\left( t\right) .$ We provide a more detailed description of the general equilibrium structure in “Appendix 2”.

## Impact of Medical Progress

### Demand for Health Care and Value of Life (VOL)

In “Appendix 4” we show that the impact of medical progress, as measured by an increase in the level of medical technology, $dM>0,$ on the demand for health care at $\left( a,t\right)$ is described by

25 $$\begin{aligned} \frac{dh\left( a,t\right) }{dM}=\underbrace{\frac{-\mu _{hM}}{\mu _{hh}}}_{ \text { (i) }}+\underbrace{\frac{\mu _{h}\left( a,t\right) }{\mu _{hh}}}_{{<0} }\Bigg (\frac{1}{p_{H}\left( t\right) }\underbrace{\frac{dp_{H}\left( t\right) }{dM}}_{\text {(ii)}}-\frac{1}{\psi \left( a,t\right) }\underbrace{ \frac{d\psi \left( a,t\right) }{dM}}_{\text {(iii)}}\Bigg ). \end{aligned}$$

Term (i) represents the effect of medical technology on the demand for health care through changes in the effectiveness of care. If technology raises the marginal effectiveness of health care ($\mu _{hM}<0$), term (i) is positive and more health care will be consumed at $\left( a,t\right)$ in response to medical progress. Note, however, that some technologies, described by $\mu _{hM}\ge 0$ may effectively replace intensive health care and, thus, lead to the opposite impact on the demand for healt care. Term (ii) implies that the demand for health care tends to fall if medical progress raises its price. Finally, the demand for health care changes in line with the impact of medical progress on the VOL [term (iii)].

The impact of medical progress on the VOL can be written as

26 $$\begin{aligned}&\frac{d\psi \left( a,t\right) }{dM}\nonumber \\&\quad =\int _{a}^{\omega }R({\widehat{a}},a)\Bigg ( -v\left( {\widehat{a}},t+{\widehat{a}}-a\right) \underbrace{\int _{a}^{{\widehat{a}} }\frac{dr\Big (t+\hat{{\hat{a}}}-a\Big )}{dM}d\hat{{\hat{a}}}}_{\text { (iii.i) }} +\underbrace{\frac{dv\left( {\widehat{a}},t+{\widehat{a}}-a\right) }{dM}}_{\text { (iii.ii) }}\Bigg )d{\widehat{a}}\nonumber \\ \end{aligned}$$

where $v\left( {\widehat{a}},t+{\widehat{a}}-a\right)$ and $R({\widehat{a}},a)$ are given by (15) and (16), respectively, and where

27 $$\begin{aligned} \frac{dv\left( {\widehat{a}},t+{\widehat{a}}-a\right) }{dM}=\left( 1-\frac{ uu_{cc}}{u_{c}^{2}}\right) \frac{dc\left( {\widehat{a}},t+{\widehat{a}}-a\right) }{dM}. \end{aligned}$$

Thus, technology bears on the VOL through two channels: through changes in the interest rate at which the monetary value of each remaining life year is discounted [term (iii.i)], and through changes in age-specific consumption over the remaining life-course [term (iii.ii) and (27)]. According to (iii.i), the VOL increases whenever improvements in medical technology reduce the interest rate, an effect that arises only in general equilibrium. Noting that $1-\frac{uu_{cc}}{u_{c}^{2}}>0$ (see “Appendix 4”), term (iii.ii) implies that a positive effect of medical technology on future consumption translates into an increase in the demand for health care.

Generally, we can write $c\left( {\widehat{a}},t+{\widehat{a}}-a\right) =c\left( a,t\right) \exp \left[ \int _{a}^{{\widehat{a}}}g_{c}(\hat{{\hat{a}}},t+\hat{\hat{ a}}-a)d\hat{{\hat{a}}}\right]$, where $c\left( a,t\right)$ is the initial consumption level at birth, and where

$$\begin{aligned} g_{c}\Big (\hat{{\hat{a}}},t+\hat{{\hat{a}}} -a\Big ):=\frac{u_{c}}{u_{cc}c\Big (\hat{{\hat{a}}} ,t+\hat{{\hat{a}}}-a\Big )}\left[ \rho -r\Big (t+ \hat{{\hat{a}}}-a\Big )+\mu \Big (\hat{{\hat{a}}},t+ \hat{{\hat{a}}}-a\Big )\right] \end{aligned}$$

is the rate of consumption growth at $(\hat{{\hat{a}}},t+\hat{{\hat{a}}}-a)$ as given by the dynamic Euler equation (17). Thus, we have

28 $$\begin{aligned}&\frac{dc\left( {\widehat{a}},t+{\widehat{a}}-a\right) }{dM}\nonumber \\&\quad =c\left( {\widehat{a}} ,t+{\widehat{a}}-a\right) \left\{ \frac{1}{c\left( a,t\right) }\frac{dc\left( a,t\right) }{dM}+\int _{a}^{{\widehat{a}}}\frac{dg_{c}(\hat{{\hat{a}}},t+\hat{ {\hat{a}}}-a)}{dM}d\hat{{\hat{a}}}\right\} , \end{aligned}$$

according to which the impact of medical progress on consumption at $\left( {\widehat{a}},t+{\widehat{a}}-a\right)$ is governed by two possibly offsetting effects: the impact on initial consumption $c\left( a,t\right)$, which is implicitly determined through the life-cycle budget constraint, and the impact on the growth rate of consumption over the life-cycle, the latter of which depends in particular on changes in the interest rate and the mortality rate. More specifically, medical change tends to increase the growth rate of consumption at $(\hat{{\hat{a}}},t+\hat{{\hat{a}}}-a)$ to the extent that it increases the spread between interest rate and mortality rate $r(t+\hat{{\hat{a}}}-a)-\mu (\hat{{\hat{a}}},t+\hat{{\hat{a}}}-a),$ e.g. by lowering mortality.

Given the offsetting effects in (25)–(28) it is difficult to arrive at a general statement about the impact of medical technology on the VOL and on the demand for health care without placing undue restrictions on the model. At this point, we therefore content ourselves with having identified the various channels through which medical progress impacts consumption and the demand for health care and defer a quantitative assessment of the various offsetting effects to our numerical analysis in Sect. 6.3.

### Prices

In the following, we assume that the production in the final goods and health care sector, respectively, is described by the set of Cobb–Douglas production functions

29 $$\begin{aligned} Y\left( t\right) &= K_{Y}\left( t\right) ^{\alpha }\left[ A\left( t\right) L_{Y}(t)\right] ^{1-\alpha } \end{aligned}$$

30 $$\begin{aligned} F\left( t\right) &= K_{H}\left( t\right) ^{\beta }\left[ L_{H}(t)\right] ^{1-\beta }, \end{aligned}$$

with $\alpha ,\beta \in \left[ 0,1\right]$. Noting from “Appendix 3” that all prices in the economy can be calculated as a function of the interest rate, we show in “Appendix 4” that

31 $$\begin{aligned} \frac{dw\left( t\right) }{dM} &= -\frac{\alpha }{1-\alpha }\frac{w\left( t\right) }{r\left( t\right) +\delta }\frac{dr\left( t\right) }{dM}, \end{aligned}$$

32 $$\begin{aligned} \frac{dp_{H}\left( t\right) }{dM} &= \frac{p_{H}\left( t\right) }{r\left( t\right) +\delta }\frac{\beta -\alpha }{1-\alpha }\frac{dr\left( t\right) }{ dM}, \end{aligned}$$

The general equilibrium impact of medical progress on the wage rate as well as on the price for health care is thus determined by its effect on the market interest rate. Most importantly, the impact of medical change on the wage rate is always opposite to its impact on the interest rate. This is because a reduction (increase) in the market interest rate leads to an increase (reduction) of capital employed in production which translates into an increase (decrease) in the marginal productivity of labour. The effect of medical progress on the price of health care is ambiguous. As Eq. (32) indicates, we have $sgn\frac{dp_{H}\left( t\right) }{dM}=-sgn \frac{dr\left( t\right) }{dM}$ if and only if $\beta <\alpha$, i.e. if and only if the capital elasticity is lower in the health care sector as compared to the remaining industry. In Sect. 6.1 we will provide empirical evidence to the effect that this is, indeed, the case. Whenever medical change induces a reduction in the interest rate, the corresponding boost to the wage rate drives up the price for health care, the latter being produced in a relatively labour intensive way.

While we are unable to present a closed theoretical expression for the effect of medical progress on the interest rate, $\frac{dr\left( t\right) }{dM}$, we can draw on the mechanics of the capital market to derive some insight into the matter. Denote by $K_{Y}^{d}\left( t,r\right)$ and $K_{H}^{d}\left( t,r\right)$ the capital demand functions in the final goods and health care sector, respectively. From (20) and (22) it is readily checked that, ceteris paribus, capital demand decreases in the interest rate, *r*, and does not directly depend on the state of medicine, *M*. In contrast, the supply of capital $K^{s}\left( t,r,M\right)$ can be shown to increase, ceteris paribus, with both *r* and *M*. Denote by $r\left( t\right)$ the interest rate that equilibrates the capital market such that $K_{Y}^{d}\left( t,r\left( t\right) \right) +K_{H}^{d}\left( t,r\left( t\right) \right) =K^{s}\left( t,r\left( t\right) ,M\right)$ in period *t* and consider now an improvement in medical technology, $dM>0.$ While it is difficult to assess the general equilibrium impact, it is easy to see that the instantaneous impact involves an outward shift of the capital supply function and, thus, $K_{Y}^{d}\left( t,r\left( t\right) \right) +K_{H}^{d}\left( t,r\left( t\right) \right) <K^{s}\left( t,r\left( t\right) ,M+dM\right)$. The excess supply of capital then implies a downward pressure on the interest rate, $\frac{dr\left( t\right) }{dM}<0,$ and a boost to earnings and the price of health care, $\frac{ dw\left( t\right) }{dM}>0$ and $\frac{dp_{H}\left( t\right) }{dM}>0.$ This intuition is, indeed, confirmed by the numerical analysis in Sect. 6.3.

### Economic Performance (GDP)

Finally, consider the impact of medical progress on the GDP per capita as a measure of economic performance. Note that in our framework GDP is defined as the sum of output in the final goods and health care sector, as measured in units of the final good, $GDP\left( t\right) =Y\left( t\right) +p_{H}\left( t\right) F\left( t\right) .$ Expressing GDP per capita

$$\begin{aligned} \frac{GDP\left( t\right) }{N\left( t\right) }=\frac{L\left( t\right) }{ N\left( t\right) }\frac{GDP\left( t\right) }{L\left( t\right) } \end{aligned}$$

as the product of the employment rate $\frac{L\left( t\right) }{N\left( t\right) }$ and the GDP per worker $\frac{GDP\left( t\right) }{L\left( t\right) }$, it is easy to see that the impact of medical progress on economic performance comes (1) through a change in the employment rate; and (2) through a change in the GDP per worker. The impact of medical innovation on the employment rate strongly depends on the age-profile of mortality rates and their dependency on medical progress. While the dependency is generally ambiguous, we would conjecture that in developed economies in which technology-related gains in survival are concentrated amongst the older population, the likely impact of medical progress on the employment rate is negative, and this is, indeed, confirmed by our numerical simulation calibrated to a US setting.

In “Appendix 4” we show that for the Cobb–Douglas functions in (29) and (30) we can write the equilibrium level of GDP per worker as a function of the employment share $\lambda \left( t\right) :=L_{Y}\left( t\right) /L\left( t\right)$ and the aggregate capital intensity $K\left( t\right) /L\left( t\right)$

33 $$\begin{aligned}&\frac{GDP\left( t\right) }{L\left( t\right) } \nonumber \\&\quad =\frac{Y\left( t\right) +p_{H}\left( t\right) F\left( t\right) }{L\left( t\right) }=\left[ 1+\frac{ p_{H}\left( t\right) F\left( t\right) }{Y\left( t\right) }\right] \frac{ Y\left( t\right) }{L\left( t\right) } \nonumber \\&\quad =\frac{1-\alpha +\left( \alpha -\beta \right) \lambda \left( t\right) }{ 1-\beta }A\left( t\right) ^{1-\alpha }\left[ \frac{\alpha \left( 1-\beta \right) }{\beta \left( 1-\alpha \right) +\left( \alpha -\beta \right) \lambda \left( t\right) }\right] ^{\alpha }\left( \frac{K\left( t\right) }{ L\left( t\right) }\right) ^{\alpha }. \end{aligned}$$

Taking the total differential of this expression with respect to *M* we can then show that (see “Appendix 4”)

34 $$\begin{aligned}&\frac{d}{dM}\left( \frac{GDP\left( t\right) }{L\left( t\right) }\right) \nonumber \\&\quad = \frac{-\left( 1-\alpha \right) \left( \alpha -\beta \right) ^{2}\left[ 1-\lambda \left( t\right) \right] }{\left[ 1-\alpha +\left( \alpha -\beta \right) \lambda \left( t\right) \right] \left[ \beta \left( 1-\alpha \right) +\left( \alpha -\beta \right) \lambda \left( t\right) \right] }\frac{ GDP\left( t\right) }{L\left( t\right) }\frac{d\lambda \left( t\right) }{dM} \nonumber \\&\qquad +\alpha \frac{GDP\left( t\right) }{K\left( t\right) }\frac{d}{dM}\left( \frac{K\left( t\right) }{L\left( t\right) }\right) . \end{aligned}$$

It is readily verified that $\frac{d}{dM}\left( \frac{GDP\left( t\right) }{L\left( t\right) }\right) >0$ holds if $\frac{d\lambda \left( t\right) }{dM}\le 0$ and $\frac{d}{dM}\left( \frac{K\left( t\right) }{ L\left( t\right) }\right) \ge 0.$ Thus, medical progress tends to raise GDP per worker (1) if, for a given structure of the economy as described by the employment share $\lambda \left( t\right) ,$ it leads to capital deepening, i.e. to an increase in the economy-wide capital intensity $\frac{K\left( t\right) }{L\left( t\right) };$ and (2) if it induces a shift in resources to the more labour intensive health care sector, as measured by a decline in the employment share of final goods production $\lambda \left( t\right) .$^19^ Our numerical analysis in Sect. 6.3 shows that, indeed, medical innovation triggers both an increase in the aggregate capital stock per worker and a reduction in final goods employment. Thus, its impact on the GDP per worker is unambiguously positive. Whether or not this induces an increase in GDP per capita then depends on the extent to which the employment rate $L\left( t\right) /N\left( t\right)$ is curbed by medical progress. For the US health care context studied in Sect. 6.3, we find the increase in the GDP per worker to be the (weakly) dominating effect.

## Numerical Analysis

To gain a more quantitative understanding of the channels through which medical progress bears on the economy we now resort to a numerical analysis. For this purpose we calibrate the benchmark steady state of the model to reflect the US economy in the year 2003. We then study in a quasi experimental way the impact of an (unanticipated) medical innovation that increases the effectiveness of health care in lowering mortality.^20^

### Specification of the Numerical Analysis

The main components of our numerical model are specified as follows.

#### Demography

With model time progressing in single years, individuals enter the model economy at age 20 and can live up to a maximum age 100.^21^ In our model, a “birth” at age 20 implies that $\omega =80$. Population growth is partly endogenous due to endogenous mortality and partly exogenous due to a fixed growth rate of the number of births $\log (\frac{B_t}{B_{t-1}}) = \nu =0.013$, which is calibrated to match the elderly share of the adult (20 years and older) US population, equalling $17.6\%$ according to the decennial census in the US in the year 2000.^22^ Due to the exogenous path of births, our results are not confounded by a variation in birth numbers across the scenarios.

#### Mortality

**Fig. 1:**
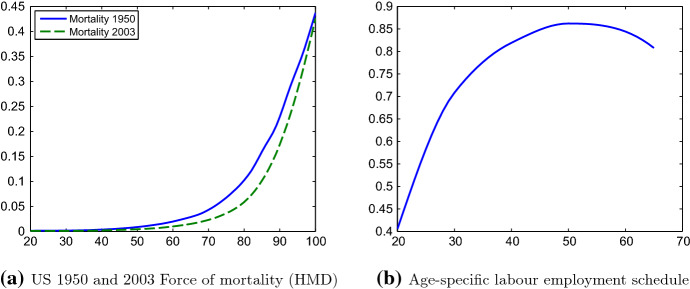
Mortality and labour employment age-profiles. (Color figure online)

The force of mortality, $\mu$, is endogenously determined in the model, depending on health care, *h*, as a decision variable; an exogenous level of medical technology, *M*; and an exogenous age-dependent base mortality, ${\widetilde{\mu }}\left( a\right)$. As not all reductions in mortality can be attributed to health expenses or technological progress (see e.g. Hall and Jones 2007), we introduce an exogenous factor *I*(*a*) that captures changes in age-dependent mortality rates due to exogenous circumstances. Generalising Kuhn et al. (2011, 2015) we formulate

$$\begin{aligned} \mu (a,t)={\widetilde{\mu }}(a)\cdot \left( I(a)-\eta (a)\left[ h(a,t)\cdot M(t)\right] ^{\epsilon (a)}\right) , \end{aligned}$$

where $\eta (a)$ is a parametric function reflecting decreasing efficiency of health care with age, and where $\epsilon (a)$ reflects the age-specific elasticity of mortality with respect to health care as reported in Hall and Jones (2007). For the base mortality ${\widetilde{\mu }} (a)$ we employ age-specific mortality rates for the year 1950 in the US, as reported in the Human Mortality Database (HMD) (see Fig. 1a). The age-dependent parametric functions $\eta (a)$ and *I*(*a*) are then chosen to approximate age-specific health expenditures and mortality $\mu (a,t)$ in the year 2003.^23^ We normalise the state of medical technology to the year 2003 and, thus, set $M(t)\equiv 1$ in the benchmark case.

#### Utility

We assume instantaneous utility to be given by

$$\begin{aligned} u(a,t)=b+\frac{\left( c(a,t)-c_{0}\right) ^{1-\sigma }}{1-\sigma }, \end{aligned}$$

where $$c_{0}=\$11{,}000$$ is an exogenous minimal consumption level.^24^$$^{,}$$^25^ We choose the inverse of the elasticity of intertemporal substitution to be $$ \sigma =1.75$$ which is within the range of empirically consistent values, as suggested by Chetty (2006). Setting $$b=8$$ then guarantees that $$u(a,t)\ge 0$$ throughout. The associated VOL lies within the range of plausible estimates, as suggested in Viscusi and Aldy (2003). Finally, we assume a rate of time preference $$\rho =0.02$$.

#### Effective Labour Supply and Income

We construct the effective supply of labour ${\hat{l}}\left( a\right)$, as depicted in Fig. 1b, from age-specific earnings data for the year 2003 that is provided by the Bureau of Labor Statistics (BLS) in their Current Population Survey (CPS). We rescale the earnings schedule such that the employment-population ratio *L*(*t*)/*N*(*t*) matches the empirical value of 62% for the US in 2003, as reported by the BLS. Individuals aged 65 or older are assumed to have no income from labour but receive a fixed social security pension for the remainder of their lifetime, as detailed further on below.

#### Production

Production of the final good is described by Eq. (29), where labour productivity, *A*(*t*), is calibrated so that ${\hat{l}}(50)w(t)$ matches the average earnings of a 50-year old in 2003; and where the elasticity of capital $\alpha$ is chosen to be 1/3.

The health care sector produces medical goods and services that individuals purchase with a view to lowering their mortality. The production function is given by Eq. (30). For the production elasticity of capital in the health care sector we take an estimate from Acemoglu and Guerrieri (2008) and set $\beta =1/5$. Finally, we assume a rate of capital depreciation equal to $\delta =0.05.$

#### Health Insurance, Medicare and Social Security

Health expenditures are subsidised through two different channels: (a) private health insurance with coinsurance rate $$\phi _{P}$$ and (b) Medicare for the elderly (available after retirement) with coinsurance rate $$\phi _{MC}$$. Private health insurance is financed through a “risk-adequate” premium equal to the expected health expenditure covered by the insurance for an individual at a given time and age. It is thus given by $$\tau _{P}= \left[ 1-\phi _{P}(a,t)\right] p_{H}(t)h^{*}(a,t)$$, where $$h^{*}(a,t) $$ denotes the equilibrium demand for health care at (*a*, *t*). Following Zhao (2014) we assume that 70% of the US workforce is health insured, with 70% of expenses being covered (in 2000). Thus, we assume that 51% of health expenditures are paid out-of-pocket on average among the working population. Zhao (2014) states that 35% of the elderly have health insurance with a coverage of 30%, leading to average health insurance subsidies of 10.5%. We assume that Medicare covers 38 % of the health expenses of the elderly.^26^ This results in 51.5% out-of-pocket expenditures for the elderly. In total, the out-of-pocket share of health expenses paid by the individual is

$$\begin{aligned} \phi (a,t) =\left\{ \begin{array}{ll} 0.51&{}{\text { if }}a<a_{R} \\ 0.515&{}{\text { if }}a\ge a_{R}, \end{array} \right. \end{aligned}$$

where $a_{R}=65$ is the mandatory age of retirement. Medicare is financed through a payroll tax, with the rate ${\hat{\tau }}_{MC}$ being endogenously determined such that the Medicare budget constraint

$$\begin{aligned} \int _{a_{R}}^{\omega }\left[ 1-\phi _{MC}(a,t)\right] p_{H}\left( t\right) h^{*}(a,t)N(a,t)da={\hat{\tau }}_{MC}(t)w(t)L(t), \end{aligned}$$

holds.

Social security, received by retirees, is financed through a payroll tax which is determined endogenously from the social security budget constraint

$$\begin{aligned} \int _{a_{R}}^{\omega }\pi (a,t)N(a,t)da={\hat{\tau }}_{\Pi }(t)w(t)L(t), \end{aligned}$$

where $$\pi (a,t)$$ is the social security pension and $${\hat{\tau }}_{\Pi }$$ the payroll tax devoted to social security. We assume social security benefits to be exogenous and use the CPS Annual Social and Economic Supplement data for the year 2003 which quotes a mean social security income of approximately $10,300 for individuals aged 65 years or older. Thus, we set $$\pi (a,t)=\$10{,}300$$ for $$a\ge 65$$ and to zero otherwise.

Altogether, individuals face the following taxes (including the premium for the private health insurance):

$$\begin{aligned} \tau (a,t)=\underbrace{{\hat{\tau }}_{\Pi }(t)l(a)w(t)}_{=\tau _{\Pi }(a,t)}+ \underbrace{\underbrace{{\hat{\tau }}_{MC}(t)l(a)w(t)}_{=\tau _{MC}(a,t)}+ \underbrace{\left[ 1-\phi _{P}(a,t)\right] p_{H}(t)h^{*}(a,t)}_{\tau _{P}(a,t)}}_{=\tau _{H}(a,t)}. \end{aligned}$$

#### Overview of Functional Forms and Parameters

Table 1 summarises the functional forms and parameters we are employing. Table 2 shows further parameters and functional forms that are used in the calibration to match various empirical moments. The $\equiv$ symbol denotes that the function is assumed to be constant in all arguments.

**Table 1:** Parameters and functional forms

Parameter and functional forms	Description
$\omega = 80$	Life span
$\rho = 0.02$	Pure rate of time preference
$\sigma = 1.75$	Inverse elasticity of intertemporal substitution
$c_{0}=\$11{,}000$	Subsistence minimum
$a_{R} = 65$	Mandatory retirement age
$\delta = 0.05$	Depreciation rate
$\alpha = 1/3$	Elasticity of capital in *Y*
$\beta = 1/5$	Elasticity of capital in *F*
$u(a,t)=b+\frac{(c(a,t)-c_{0})^{(1-\sigma )}}{1-\sigma }$	Instantaneous utility function
$B(t)=B_{0}\exp [\nu t]$	Number of births
$s(t)=\frac{1}{N(t)}\int _{0}^{\omega }\mu (a,t)k(a,t)N(a,t)da$	Transfer from accidental bequests
$Y(t)=K_{Y}(t)^{\alpha }(A(t)L_{Y}(t))^{(1-\alpha )}$	Production in manufacturing sector
$F(t)=K_{H}(t)^{\beta }(L_{H}(t))^{1-\beta }$	Production in health sector
$\mu (a,t)={\widetilde{\mu }}(a) \left( I(a)-\eta (a)\left[ h(a,t) M(t)\right] ^{\epsilon (a)}\right)$	Mortality rate
$\phi \left( a,t\right) = \left\{ 0.51\text { if }a<65,\ 0.515\text { if } a\ge 65 \right\}$	Total coinsurance

**Table 2:** Moments to match

Parameter and functional forms	Description	Moments to match
$$b = 8$$	Constant offset in utility function	Value of life
$$\nu = 0.013 $$	Growth rate of birth numbers	Population share of 65 years and older
*I*(*a*)	Exogenous impacts on mortality	Life-expectancy
$$\epsilon (a)$$	Concavity in mortality function	Age-specific health expenditures
$$\eta (a)$$	Effectiveness of health care	Age-specific health exp. and life-expectancy
$$M(t)\equiv 1$$	Medical technology	Aggregate health exp. and life-expectancy
$$A(t)\equiv 2.995$$	Manufacturing technology	GDP per capita
$$\pi \left( a,t\right) =\{ 0\text { if }a<65$$, $10,300 if $$a\ge 65 \}$$	Pension	Social security
$$\phi _{P}\left( a,t\right) =\left\{ 0.51\text { if }a<65,\ 0.895\text { if }a\ge 65 \right\} $$	Age-specific private coinsurance	Data in Zhao (2014)
$$\phi _{MC}\left( a,t\right) =\left\{ 1\text { if }a<65,\ 0.62\text { if } a\ge 65 \right\} $$	Age-specific medicare coinsurance	Data in Zhao (2014)

A detailed description of the solution of the numerical problem is provided in “Appendix 5”.

### Benchmark

**Fig. 2:**
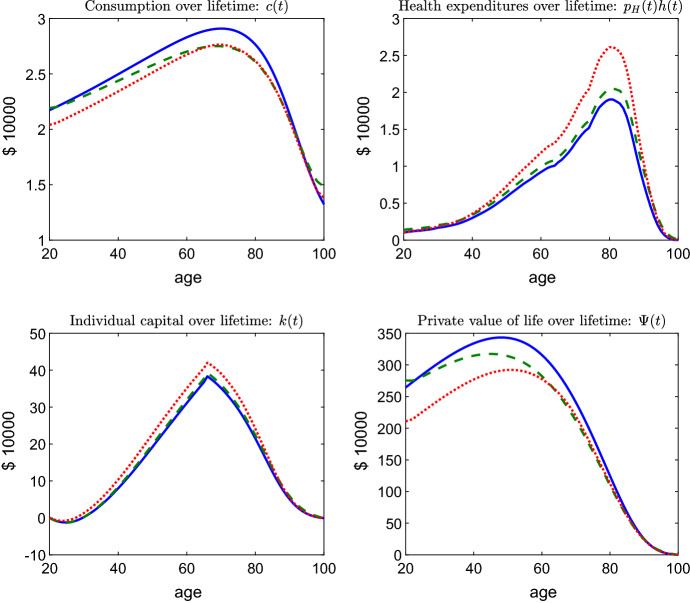
Consumption, health expenditure, asset and value of life profiles for the benchmark (blue, solid line), for the medical advance in general equilibrium (green, dashed line), and for the medical advance in partial equilibrium (red, dotted line). (Color figure online)

**Fig. 3:**
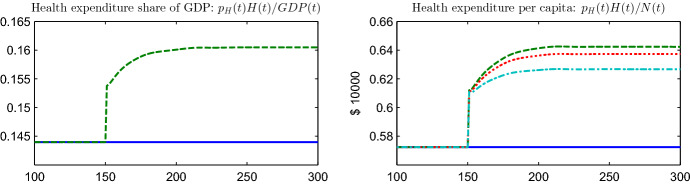
Health expenditure share of GDP (left panel) and health expenditure per capita (right panel) for the benchmark (blue, solid line) and for the unanticipated increase in *M* in general equilibrium (green, dashed line). The cyan, dashed-dotted line indicates the pure shift in individual demand, $h\left( a,t\right) ,$ holding the population shares, $N\left( a,t\right) /N\left( t\right) ,$ and the price of medical care, $p_{H}\left( t\right) ,$ constant. The red, dotted line denotes the effect holding only $p_{H}\left( t\right)$ constant. (Color figure online)

**Fig. 4:**
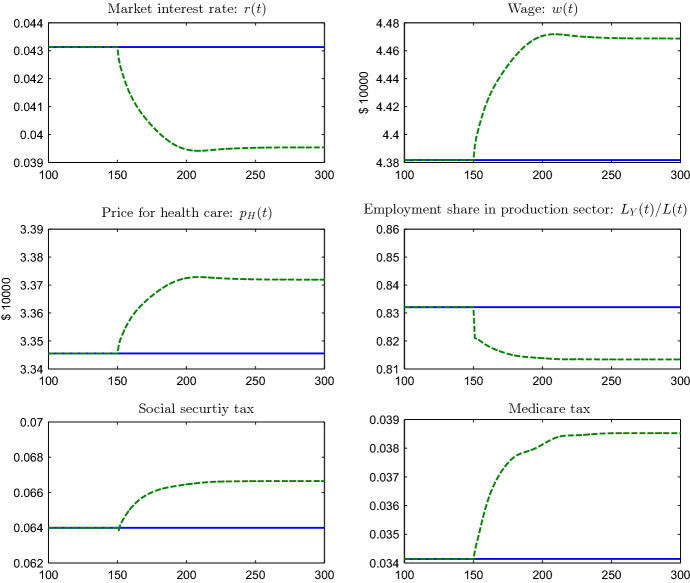
Market prices, employment share and taxes. (Color figure online)

In order to economise on space we illustrate the benchmark allocation in the same graphs as our experiment (see Figs. 2, 3, 4). The benchmark allocation is depicted by blue, solid plots throughout, whereas the experiment is depicted by green, dashed plots. Figure 2 also contains red, dotted plots, which refer to a partial equilibrium allocation.

At the level of the individual life-cycle, the salient features of the benchmark allocation can be summarised as follows. Consumption is hump-shaped (see Fig. 2, upper left panel). The fact that the interest rate (approx. 4.3%) lies above the rate of time preference (2%) implies a rising consumption until around age 70. Due to missing annuity markets, consumption falls, however, at higher ages as implied by the individual Euler equation (17).^27^ Individual health expenditures follow a hump-shaped pattern (Fig. 2, upper right panel). While the demand for care grows very moderately up to age 40, it exhibits from then on a strong increase up to age 80 before dropping again for the highest ages. Note that such a pattern is consistent with recent evidence in Martini et al. (2007) and De Nardi et al. (2016).^28^ In order to finance the significant co-payments involved with the high levels of health expenditure in old age, the individual accumulates a considerable stock of assets (Fig. 2, lower left panel).^29^

The value of life (VOL) peaks at approx. age 50 (Fig. 2, lower right panel), which is consistent with empirical evidence on the value of a statistical life in Aldy and Viscusi (2008). In our model, the hump-shaped age-profile of the VOL follows the equally hump-shaped age profile of individual consumption. In line with (19), the VOL increases during early life where consumption levels are low such that the value of life years written off falls short of a high return on the VOL. This relationship reverses in old age. The remaining life expectancy at age 20 is 58.0 years in the benchmark case and, thus, matches the empirical value for the US in 2003 (58.1 years, HMD) very well.

GDP per capital amounts to $39,700 [$39,700 according to Table 1.5.5 of the revised National Income and Product Accounts of the Bureau of Economic Analysis (BEA), 2003], and health expenditures per capita amount to $5720 [$5750 according to NHEA, 2003]. The health share (in GDP) in the benchmark case is 14.4% and matches the data from the National Health Expenditure Accounts provided by CMS.^30^ Furthermore, the benchmark model features a Medicare share of 2.3% [2.3% according to Zhao (2014)]. Finally, while our calibration strategy involves the matching of the population share 65 years and older and the employment-population ratio as key demographic indicators, we note the good incidental fit with the capital-output ratio, the interest rate, the wage rate and the Medicare tax as key economic indicators. Table 3 summarises how the benchmark model fits the data.

**Table 3:** Fit of the benchmark model (data provided for the year 2003) and outcomes for an unanticipated medical advance

Name	Data	Benchmark	Medical advance
GDP per capita	$39,700	$39,700	$40,000
Health spending per capita	$5750	$5720	$6420
Health spending (% of GDP)	14.4%	14.4%	16.0%
Medicare expenditures (% of GDP)	2.3%	2.3%	2.7%
Life expectancy at age 20	58.1	58.0	59.5
Population share 65 years and $$\hbox {older}^{\rm a}$$	17.6%	17.5%	18.4%
Employment-population $$\hbox {ratio}^{\rm a}$$	62%	62%	61.5%
Capital-output $$\hbox {ratio}^{\rm b}$$	3.1	3.3	3.5
Interest rate	$$4.12\%^{\text {c}}$$	4.3%	3.95%
Annual earnings (full-time) ^d^	$42,201	$43,800	$44,700
Medicare payroll tax rate, $${\hat{\tau }}_{MC}$$	2.9%	3.4%	3.8%

$^\mathrm{a}$The population share of individuals aged 65 or older as well as the employment-population ratio refers to the total population aged 20 or older

$^\mathrm{b}$The capital-output ratio was calculated as the ratio of the capital stock and the gross domestic product as provided in the National Income and Production Accounts of the Bureau of Economic Analysis (BEA) in 2003. In the model it is calculated as *K*(*t*)/*GDP*(*t*)

$^\mathrm{c}$Average of the monthly prime loan rates for the year 2003 as reported by the Federal Reserve Bank of St Louis (https://fred.stlouisfed.org/series/MPRIME)

$^\mathrm{d}$Average annual full-time earnings for the year 2003 as reported in the OECD employment statistics (https://stats.oecd.org/Index.aspx?DataSetCode=AV_AN_WAGE)

A clarifying remark is warranted on the purpose and design of our numerical analysis. The main objective lies in an analytical and quantitative understanding of the mechanisms which are underlying the macroeconomic transmission of medical change. In order to avoid that these are confounded by other sources of change, we have structured our numerical analysis in a way that the economy is “quasi-stationary” in the years surrounding a medical technology shock. This is why we are abstracting from time-trends in the states of technology, $A\left( t\right)$ and $M\left( t\right)$, in the growth rate of the nuber of births, $\nu ,$ and in the policy variables, $\phi \left( a,t\right)$ and $\pi \left( a,t\right)$.^31^ Nevertheless, we have calibrated the model to the US economy in the year 2003 in order to provide a realistic static backdrop for our numerical experiment.

### Impact of Medical Progress

Considering the model time frame from $t=100$ to $t=300$, we study the impact of an unanticipated increase in the state of the medical technology from $M\left( t\right) =1$ for $t\le 150$ to $M\left( t\right) =2$ for $t>150.$^32^ The advance of medical technology renders health care more effective in lowering mortality.^33^

Based on a comparison of steady-state values, we find that the innovation raises the remaining life-expectancy of a 50 year old by some 1.1 years and induces additional (discounted) expenditures of about $18,500 over the remaining life-course. These magnitudes are broadly in line with evidence provided by Cutler (2007) on the impact of revascularisation, as was introduced into the US during the late 1980s. Cutler finds that for a patient with myocardial infarction, revascularisation would raise life-expectancy by about 1 year and induce about $40,000 in additional expenditure. While the impact of innovation in our model is, thus, comparable in the order of magnitude, it should be borne in mind that the figures are not directly comparable, as in Cutler (2007) the values apply (ex-post) to individuals who have had a heart attack, whereas in our model they apply (ex-ante) to the representative agent on whom we are building our macroeconomic analysis.

When considering the life-cycle outcomes for a representative individual born into a steady-state economy with the more effective medical technology, we find the following effects: As Fig. 2 (upper panels) illustrates, the innovation induces individuals to reallocate expenditure from consumption to health care. Indeed, the drop in consumption is persistent over the life-cycle but the highest ages, where the lower mortality risk induces individuals to raise consumption. When it comes to the impact of the innovation on the demand for health care (as measured by individual health expenditure), a more ambiguous picture emerges: For a given set of prices, the expenses for medical care would increase for all age groups by a substantive amount (see the red, dotted plot). However, such a partial equilibrium take is inappropriate, as the general equilibrium impact of the innovation on the underlying demand and supply system needs to be taken into account. Once we do this, much of the demand expansion vanishes (see green, dashed plot). This notwithstanding, the medical innovation raises remaining life-expectancy at age 20 from 58.0 to 59.5 years. Notably, the strong increase in demand for a constant set of prices would induce an additional gain of only 0.35 life years. The finding that gains to life-expectancy arise from medical progress itself rather than from the ensuing boost in health care utilisation is consistent with recent empirical evidence from Skinner and Staiger (2015) who show that the marginal returns to medical spending are very low once the state of medicine is controlled for.

Equation (25) affords some insight into the demand response of individual health care to medical progress. Obviously, the increased marginal effectiveness of health care through medical progress ($\mu _{hM}<0$) boosts demand, an effect that is consistent with the empirical evidence in Baker et al. (2003), Cutler and Huckman (2003), Wong et al. (2012) and Roham et al. (2014).^34^ The effect is dampened, however, by the ensuing reduction in consumption over the remaining life-time, which tends to diminish the VOL (but within the highest age groups) and, thus, the individual’s willingness to pay for health care. Notably, the consumption level drops because a greater part of the life-cycle budget is allocated to health care and because the remaining budget now needs to be spread over a longer life-time. According to Eq. (28), however, improved survival chances also induce individuals to shift consumption into higher age classes, a force that leads to increasing consumption at the highest ages.

Overall, the reallocation of resources from consumption to health care in response to medical progress is substantial in a partial equilibrium context. In general equilibrium, it is subject, however, to additional impacts from the price changes induced. Most notably, medical progress triggers a reduction in the market interest rate *r* and an increase in the price for health care $p_{H}$ (which will be discussed later). While the reduction in the market interest rate works to increase the value of life and, thus, to boost the demand for health care, the negative impact of the price increase dominates and dampens the demand increase in general equilibrium.^35^ We find that while per capita health care expenditure would increase by some 30% in partial equilibrium, in general equilibrium it increases by only 12.2%, and thus by less than a half.^36^ This also implies that the increase in asset holdings for the purpose of funding the additional health care is much more modest in general equilibrium (see Fig. 2, lower left panel).

We can summarise as follows:

#### Result 1

(i) Medical innovation leads to a reallocation of consumption to health care expenditures for all but the highest ages, and to a reallocation of consumption to higher ages. (ii) The general equilibrium impact of a mortality reducing medical innovation on the demand for health care tends to be dampened by an associated price increase.

Although per capita demand for health care and the associated expenditure, $p_{H}(t)H\left( t\right) /N\left( t\right)$, have increased after the innovation, (see Fig. 3, right panel) the magnitude of the effect varies across age-groups. Specifically, those over 90 exhibit a very modest demand increase in spite of the innovation. For these cohorts the willingness to pay for care, as measured by the VOL, is so low that the value of the survival gains from the innovation barely outweighs the price increase. Finally, and strikingly, the medical innovation leads to a reduction in the VOL for all but the very youngest and very oldest individuals (see Fig. 2, lower right panel). At face value, the lower willingness to pay for survival follows from the reduction in consumption over the remaining life-course.

Rewriting the first-order condition for the demand of health care (13) to $\psi \left( a,t\right) =-\phi \left( a,t\right) p_{H}\left( t\right) \mu _{h}^{-1}$, we find that the VOL is equated to the effective (or quality-adjusted) price of medical care $-\phi \left( a,t\right) p_{H}\left( t\right) \mu _{h}^{-1},$ the latter depending on both the market price and the marginal impact on mortality of health care, $-\mu _{h}$. Recalling that $\mu _{hh}>0,$ an increasing demand for care would ceteris paribus imply a greater effective price. But then it must be true that the medical innovation has lowered the effective price for medical care (recall that $\mu _{hM}<0)$ to an extent that it over-compensates the increase in the market price, $p_{H}\left( t\right) .$ Notably this finding is consistent with evidence produced by Cutler et al. (1998), Lucarelli and Nicholson (2009), Dunn (2012), Lakdawalla et al. (2015) and Hult et al. (2018) who find for a variety of treatment settings that while list prices have been subject to inflation [or stagnation in case of the anti-cholesterol drugs considered by Dunn (2012)], quality-adjusted prices have seen much lower increases, have remained constant, or have declined (in the majority of cases).^37^

From this perspective, the decline in the VOL following the medical innovation can be interpreted in terms of basic consumption theory: An optimal choice between the two goods, survival and consumption, is given if the marginal rate of substitution between survival and consumption, i.e. the VOL, equals the price of survival in terms of consumption goods, i.e. the effective price of medical care. But then a decrease in the price of survival triggers a reallocation from consumption to survival (through the purchase of additional health care), implying a decline in the marginal rate of substitution and, thus, in the VOL.

Again, we can summarise

#### Result 2

Medical innovation leads to a reduction in the VOL and in the effective (quality-adjusted) price for medical care even as it boosts the nominal price for medical care.

The innovation at $t=150$ induces an increase in the health expenditure share of the GDP by some 1.6 percentage points (Fig. 3, left panel; and Table 3). Underlying this increase in the health share is a strong increase in per capita health expenditure by some 12.2% (in the new steady state). The right panel in Fig. 3 decomposes the increase in per capita health expenditure into an increase in individual demand at each given age, $h\left( a,t\right) ,$ given the pre-innovation age-structure and price for health care (corresponding to the cyan, dashed-dotted line), the additional impact of a changing age-structure, as measured by the age-shares $N\left( a,t\right) /N\left( t\right)$ (corresponding to the distance between the cyan, dashed dotted and the red, dotted lines), and the increase in the price for health care, $p_{H}\left( t\right)$ (corresponding to the distance between the red, dotted and the green, dashed line). Overall, the instantaneous boost to demand amounts to a 6.7% increase in medical expenditure per capita (=55% of the total increase), with a further 2.8% increase following during the adjustment process (=23% of the total effect). The reason for why individual demand increases over and above the instantaneous impact lies with the fact that later born cohorts have been able to accumulate additional savings for the purchase of health care. The shift in the population structure toward higher ages with a greater demand for health care amounts to an expenditure increase by 1.8% (=15% of the total effect), with the price increase adding another 0.9% (=7% of the total effect). While a total of 78% of the increase in per capita health expenditure is, thus, explained by the boost to individual demand, induced population ageing and price inflation play a significant part over the transition phase.

The shift from final goods production to health care following the innovation leads to a reduction of the employment share in the manufacturing sector, a reduction in the interest rate and an increase in the wage rate (see Fig. 4). According to Eqs. (31) and (32) the change in the factor prices translates into an increase in the price of health care, which is underlying the dampening of the demand response to innovation.^38^ Since the increase in the price of health care is driven by changes in the factor prices and, thus, by changes in the marginal cost of producing health care, it would also arise in a setting in which the price is regulated and set in proportion to the unit cost of producing health care. Furthermore, the social security payroll tax rises, following the pronounced increase in longevity, despite the simultaneous increase in the gross wage. Similarly, Medicare payroll taxes increase as a consequence of both greater health spending and the boost in longevity.

These sectoral and price adjustments notwithstanding, the medical advance has very little impact on GDP per capita (see Table 3). The survival gains induced by the innovation are greatest among older cohorts and, for a fixed retirement age, lead to a 1% reduction in the employment-population ratio, $L\left( t\right) /N\left( t\right)$.^39^ At the same time, however, the expansion of the expected retirement period and the prospect of greater health expenditures in the presence of a more effective medical technology trigger additional savings, translating into a 4% increase in the capital stock per capita, $K\left( t\right) /N\left( t\right) .$ These channels have been confirmed empirically by Bloom et al. (2007) and De Nardi et al. (2010). Overall, this leads to capital deepening, i.e. to a higher $K\left( t\right) /L\left( t\right)$, which in optimum induces a shift of resources to the more labour intensive health care sector. As we have shown in Sect. 5, both the increase in $K\left( t\right) /L\left( t\right)$ and the shift in resources to the health care sector lead to an increase in GDP per worker. Our numerical analysis shows that for the US context we are studying, this effect is strong enough to compensate (even mildly over-compensate) the decline in the employment rate.

Thus, we can summarise the following set of insights.

#### Result 3

(i) About 78% of the increase in per capita health care expenditure following a medical innovation are due to an increase in individual demand, about 15% are due to induced population ageing, and 7% are due to a price increase. (ii) Medical innovation tends to stimulate additional saving. (iii) The ensuing increase in the economy-wide capital intensity, combined with the shift of employment into the health-care sector increase the economy-wide productivity, i.e. GDP per worker, by enough to compensate the reduction of the employment-population ratio, leading to little impact on GDP per capita.

It is worth noting that the transitional dynamics following a medical innovation tie in closely with recent findings about the impact of capital deepening on the structural composition of an economy. Acemoglu and Guerrieri (2008) show for a two-sector economy that capital deepening, i.e. an increase in the economy-wide capital intensity tends to raise the output share of the capital-intensive sector but also induces a shift of both labour and capital inputs into the labour intensive sector. These shifts are accompanied by an increase in the wage rate, as is the case in our model. Acemoglu and Guerrieri (2008) go on to show that the same process is underlying unbalanced growth whenever productivity growth is larger in the capital-intensive sector (see also Baumol 1967).

While the transition to a new equilibrium after a medical innovation in our model follows a similar process, this is for rather different reasons. First, technical progress occurs in the health care sector; second, and importantly, medical progress works through the household side of the economy: Through its impact on survival and the consequent shift of the age-structure toward older cohorts, medical progress triggers an increase in savings, and, thus, in the per capita supply of capital while at the same time reducing the per capita supply of labour. Notably, this impact is present even when holding the aggregate demand for health care fixed. As we have seen, capital deepening and the sectoral shift combine to render the overall economy more productive, as measured by GDP per worker.

## Conclusion

We have set out an OLG model built around the endogenous demand and supply of health care. In contrast to much of the received macroeconomic literature on health and health care, our model involves a rich model of the life-cycle, based on a realistic pattern of mortality. This allows us to characterise in detail the individual life-cycle allocation of consumption and health care, and to construct macroeconomic aggregates that are based on a realistic age-structure of the population. At the microeconomic level, we can study in detail how the demand for health care responds to medical progress, taking into account induced price changes and changes in the willingness-to-pay for health care, as summarised by the value of life.

Based on a calibration of the model to the US economy in the year 2003, our numerical analysis is designed to provide a quasi-experimental identification of the channels through which changes in medical technology are transmitted between individual choices and macroeconomic dynamics. Our numerical experiments yield a number of policy relevant, and potentially challenging, insights.

First, we find that a medical innovation that increases the remaining life expectancy at age 20 by some 1.5 years, boosts health expenditure per capita by some 12.2%, with 0.9 percentage points owing to price inflation, 1.8 percentage points owing to a shift in the age-structure towards older individuals with greater consumption of health care, and 9.5 percentage points owing to an increase in individual demand. Our finding that the expansion in health expenditure is mostly driven by an increase in utilisation is well in line with recent evidence (Bundorf et al. 2009; Chernew and Newhouse 2012). However, our model also suggests that in spite of its modest contribution to expenditure growth in accounting terms, the increase in the price for health care has a significant impact on demand as described in the following.

Second, more than half of the partial equilibrium impact on the individual demand for health care of a mortality reducing innovation is neutralized in general equilibrium by an increase in the price for medical care. This result indicates a need for a general equilibrium framework when it comes to assessing the impact of medical change on health care expenditure, as otherwise findings may be biased.

Third, for an economy with social security and health care organised in similarity to the US (as of 2003), a costless medical innovation does not have a negative impact on economic performance, as measured by GDP. This is despite a reduction in the employment rate due to the concentration of survival gains within the population of pensioners. The main mitigating channel is the accumulation of additional savings/capital for the purpose of financing consumption over an extended life-course and purchasing more effective health care at a higher price. Indeed, this channel is very much in line with evidence for the US on savings related to health expenditures in old age (e.g. De Nardi et al. 2010). Overall, the capital deepening of the economy combines with a shift in economic activity to the labour intensive health care sector, and translates into a higher GDP per worker. For our calibration, this effect more than compensates the decline in the employment rate. Two caveats are worth of note here: The cost of medical innovation, e.g. through the absorption of production factors within a medical R&D sector may after all induce a drag on economic growth (Jones 2016).^40^ In addition, the question as to whether additional savings are induced in the wake of a medical innovation depends on the design of the social security system. To the extent that expenditures during retirement are financed through public transfers, the savings response is weaker (Bloom et al. 2007), implying that the accumulation of additional capital may not be sufficient to offset the reduction in the employment rate. Additional offsetting impacts arise if health improvements not only translate into lower mortality but also into a greater propensity to provide labour into older ages (Kuhn and Prettner 2016).

Fourth, mortality reducing medical innovations tend to come with a reduction in the value of life over large parts of the life-course. This finding has two interesting ramifications. At face value, the reduction in the value of life arises from a reallocation by the individual of resources from consumption to health care. While per se, this is reflecting an efficient response by the individual to the availability of more effective health care, it also implies that individuals may be less willing to prevent risks to their life. Thus, some of the benefits of medical innovations in terms of improved survival prospects may well be offset by the adoption of less healthy life-styles.

Finally, the reduction in the value of life also implies a reduction in the effective (quality-adjusted) price of medical care as triggered by the innovation in spite of a parallel increase in its nominal price. This is in line with evidence for the US, as provided in Cutler et al. (1998), Lucarelli and Nicholson (2009), Dunn (2012), Lakdawalla et al. (2015) and Hult et al. (2018). Our analysis also shows that these divergent price trends are consistent with medical progress coming in the form of demand-increasing product innovation rather than process innovation.

In the present work, we have abstracted from long-run trends to productivity and population in order to avoid that these trends obfuscate the identification of the transmission channels of medical progress. Thus, we would not claim our findings to be precise in quantitative terms. This is in particular in the light of the findings by Fonseca et al. (2013) and Frankovic and Kuhn (2018, 2019) that medical progress and income growth are highly complimentary in boosting the demand for health care. We would maintain, however, that by laying out the anatomy of medical progress at the individual, sectoral and macroeconomic level our work provides a foundation for understanding the mechanics behind the quantitatively richer numerical analyses.

## Appendix 1: Optimal Solution to the Individual Life-Cycle Problem

The individual’s life-cycle problem, i.e. the maximisation of (1) subject to (2) and (5) can be expressed by the Hamiltonian

$$\begin{aligned} {\mathcal {H}}=uS-\lambda _{S}\mu S+\lambda _{k}\left( rk+lw-c-\phi p_{H}h-\tau +\pi +s\right) , \end{aligned}$$

leading to the first-order conditions

35 $$\begin{aligned} {\mathcal {H}}_{c} &= u_{c}S-\lambda _{k}=0, \end{aligned}$$

36 $$\begin{aligned} {\mathcal {H}}_{h} &= -\lambda _{S}\mu _{h}S-\lambda _{k}\phi p_{H}=0, \end{aligned}$$

and the adjoint equations

37 $$\begin{aligned} \overset{\cdot }{\lambda }_{S} &= \left( \rho +\mu \right) \lambda _{S}-u, \end{aligned}$$

38 $$\begin{aligned} \overset{\cdot }{\lambda }_{k} &= \left( \rho -r\right) \lambda _{k}. \end{aligned}$$

*Optimality conditions *(12)* and *(13): Evaluating (35) at two different ages/years $\left( a,t\right)$ and $\left( {\widehat{a}},t+{\widehat{a}}-a\right)$, equating the terms and rearranging gives us

39 $$\begin{aligned} \frac{u_{c}\left( {\widehat{a}},t+{\widehat{a}}-a\right) }{u_{c}\left( a,t\right) } &= \frac{\lambda _{k}\left( {\widehat{a}},t+{\widehat{a}}-a\right) }{\lambda _{k}\left( a,t\right) }\frac{S\left( a,t\right) }{S\left( \widehat{ a},t+{\widehat{a}}-a\right) } \nonumber \\ &= \exp \left\{ \int _{a}^{{\widehat{a}}}\left[ \rho +\mu \left( \widehat{ {\widehat{a}}},t+\widehat{{\widehat{a}}}-a\right) -r\left( t+\widehat{{\widehat{a}} }-a\right) \right] d\widehat{{\widehat{a}}}\right\} , \end{aligned}$$

which is readily transformed into the Euler equation (12) as given in the main body of the paper.

Inserting (35) into (36) allows to rewrite the first-order condition for health care as

40 $$\begin{aligned} -\mu _{h}\left( a,t\right) \frac{\lambda _{S}\left( a,t\right) }{u_{c}\left( \cdot \right) }=\phi \left( a,t\right) p_{H}\left( t\right) . \end{aligned}$$

Integrating (37) we obtain

$$\begin{aligned} \lambda _{S}\left( a,t\right) =\int _{a}^{\omega }u\left( {\widehat{a}},t+ {\widehat{a}}-a\right) \exp \left[ -\int _{a}^{{\widehat{a}}}\left( \rho +\mu \right) d\widehat{{\widehat{a}}}\right] d{\widehat{a}}. \end{aligned}$$

Using this, we can express the private VOL as

$$\begin{aligned}&\psi \left( a,t\right) :=\frac{\lambda _{S}\left( a,t\right) }{u_{c}\left( a,t\right) }\\&\quad =\int _{a}^{\omega }\frac{u_{c}\left( {\widehat{a}},t+{\widehat{a}} -a\right) }{u_{c}\left( a,t\right) }\frac{u\left( {\widehat{a}},t+{\widehat{a}} -a\right) }{u_{c}\left( {\widehat{a}},t+{\widehat{a}}-a\right) }\exp \left[ -\int _{a}^{{\widehat{a}}}\left( \rho +\mu \right) d\widehat{{\widehat{a}}}\right] d{\widehat{a}}. \end{aligned}$$

Substituting from (39) and rearranging we obtain (14) as given in the main body of the paper. Inserting this into ( 40) gives condition (13) in the main body of the paper.

*Dynamics *(17)* and *(18): Total differentiation of (35) with respect to age gives

$$\begin{aligned}&u_{cc}S\overset{\cdot }{c}+u_{c}\overset{\cdot }{S}-\overset{\cdot }{ \lambda }_{k}=u_{cc}S\overset{\cdot }{c}-u_{c}\mu S-\left( \rho -r\right) \lambda _{k} =u_{cc}S\overset{\cdot }{c}-\left( \rho -r+\mu \right) u_{c}S=0. \end{aligned}$$

From this we obtain the consumption dynamics (17) as given in the main body of the paper.

Holding prices and the state of medical technology constant, total differentiation of $-\mu _{h}\left( a,t\right) \psi \left( a,t\right) -\phi \left( a,t\right) p_{H}\left( t\right) =0$ with respect to age gives

$$\begin{aligned} -\left( \mu _{hh}\overset{\cdot }{h}+\mu _{ha}\right) \psi -\mu _{h}\overset{ \cdot }{\psi }-p_{H}\overset{\cdot }{\phi }=0. \end{aligned}$$

Substituting $p_{H}=-\mu _{h}\psi \phi ^{-1^{{}}}$ from (13) and rearranging, we obtain the dynamics for health care as reported in (18) within the main body of the paper.

## Appendix 2: Characterisation of General Equilibrium

For each period *t* we have the following unknown variables:

- inputs $$\left\{ K_{Y}\left( t\right) ,K_{H}\left( t\right) ,L_Y(t),L_H(t) \right\} ,$$
- prices $$\left\{ r\left( t\right) ,w\left( t\right) ,p_{H}\left( t\right) \right\} ,$$
- aggregate demand $$\left\{ C\left( t\right) ,H\left( t\right) \right\} , $$
- aggregate net saving, equivalent to the change in the capital stock $\overset{\cdot }{K}\left( t\right) ,$

summing up to 10 variables. These are determined through

- 4 first-order conditions on factor inputs (20)-(23), which give the factor demand functions $$\{K_{Y}^{d}\left( r,w;\,A,M,B\right) ,K_{H}^{d}\left( r,w,p_{H};\,M,B\right) ,$$$$L_Y^{d}\left( r,w;\,A,M,B\right) ,L_H^{d}\left( r,w,p_{H};\,M,B\right) \},$$ depending on prices as well as on technology and population $$\left\{ A,M,B\right\} $$ ;^41^
- a set of first-order conditions (12) and (13) for $a\in \left[ 0,\omega \right]$, which together with the individual’s life-cycle budget constraint determine the age-specific levels of consumption $c\left( a,t\right)$ and health care $h\left( a,t\right) .$ Aggregation according to (6) and (7) gives the demand for consumption $C\left( r,w,p_{H};\,M,B,\phi \right)$ and health care $H^{d}\left( p_{H};\,M,B,\phi \right) ,$ depending on the three prices as well as on technology, population and the vector of co-insurance rates;^42^
- 4 market clearing conditions
  $$\begin{aligned} K_{Y}^{d}\left( r,w;\,A,M,B\right) +K_{H}^{d}\left( r,w,p_{H};\,M,B\right) &= K, \\ L_{Y}^{d}\left( r,w;\,A,M,B\right) +L_{H}^{d}\left( r,w,p_{H};\,M,B\right) &= L(M,B), \\ F(K_{H}^{d}\left( r,w,p_{H};\,M,B\right) ,L_{H}^{d}\left( r,w,p_{H};\,M,B\right) ) &= H^{d}\left( p_{H};\,M,B,\phi \right) , \\ Y(K_{Y}^{d}\left( r,w;\,A,M,B\right) ,AL_{Y}^{d}\left( r,w;\,A,M,B\right) )) &= C\left( r,w,p_{H};\,M,B,\phi \right) \\&+\overset{\cdot }{K}+\delta K, \end{aligned}$$
  which determine the set of equilibrium prices $$\left\{ r^{*}\left( A,M,B,\phi ,\overset{\cdot }{K}\right) ,\right. $$$$\left. w^{*}\left( A,M,B,\phi ,\overset{\cdot }{K}\right) ,p_{H}^{*}\left( A,M,B,\phi ,\overset{\cdot }{K}\right) \right\} $$ and aggregate net saving, as captured by $$\overset{\cdot }{K}$$.

## Appendix 3: Equilibrium Relationships with Cobb–Douglas Technologies

Consider the Cobb–Douglas-specifications in (29) and (30). From the first-order conditions (20)–(23) we obtain the (implicit) factor demand functions

41 $$\begin{aligned} K_{Y}^{d}\left( t\right) &= \frac{\alpha Y\left( t\right) }{r\left( t\right) +\delta }, \end{aligned}$$

42 $$\begin{aligned} L_{Y}^{d}\left( t\right) &= \frac{\left( 1-\alpha \right) Y\left( t\right) }{ w\left( t\right) }, \end{aligned}$$

43 $$\begin{aligned} K_{H}^{d}\left( t\right) &= \frac{\beta p_{H}(t)F\left( t\right) }{r\left( t\right) +\delta }, \end{aligned}$$

44 $$\begin{aligned} L_{H}^{d}\left( t\right) &= \frac{(1-\beta )p_{H}\left( t\right) F\left( t\right) }{w\left( t\right) }. \end{aligned}$$

Combining (41) with (42) and (43) with (44) we obtain the equilibrium capital intensity

45 $$\begin{aligned} k_{Y}^{*}\left( t\right) :=&\frac{K_{Y}^{d}\left( t\right) }{ L_{Y}^{d}\left( t\right) }=\frac{\alpha }{1-\alpha }\frac{w\left( t\right) }{ r\left( t\right) +\delta }, \end{aligned}$$

46 $$\begin{aligned} k_{H}^{*}\left( t\right) :=&\frac{K_{H}^{d}\left( t\right) }{ L_{H}^{d}\left( t\right) }=\frac{\beta }{1-\beta }\frac{w\left( t\right) }{ r\left( t\right) +\delta }. \end{aligned}$$

and, thus, $K_{Y}^{d}\left( t\right) =k_{Y}^{*}\left( t\right) L_{Y}^{d}\left( t\right) .$ Using $k_{Y}^{*}\left( t\right)$ in (29) to rewrite $Y\left( t\right) =L_{Y}^{d}\left( t\right) A\left( t\right) ^{1-\alpha }\left( k_{Y}^{*}\right) ^{\alpha }$ and inserting this in (42) we can solve for the equilibrium wage as a function of the interest rate

47 $$\begin{aligned} w^{*}\left( t\right) ={\widehat{w}}\left( r\left( t\right) ;\,A\left( t\right) \right) =\left( 1-\alpha \right) A\left( t\right) \left[ \frac{ \alpha }{r\left( t\right) +\delta }\right] ^{\frac{^{\alpha }}{1-\alpha }}. \end{aligned}$$

This, in turn, determines the capital intensities $k_{Y}^{*}\left( t\right) ={\widehat{k}}_{Y}\left( r\left( t\right) ;\,A\left( t\right) \right)$ and $k_{H}^{*}\left( t\right) ={\widehat{k}}_{H}\left( r\left( t\right) ;\,A\left( t\right) \right)$. Using the market clearing condition $F\left( p_{H}^{*}\left( t\right) ;\,K_{H}^{*}(t),L_{H}^{*}(t)\right) =H^{d}\left( p_{H}^{*}\left( t\right) ;\,M\left( t\right) ,B\left( t\right) \right)$ together with (43) and (44) we obtain the general equilibrium price for health care as

48 $$\begin{aligned} p_{H}^{*}(t) ={\widehat{p}}_{H}\left( r(t),w^{*}\left( t\right) ,H_{d}^{*}(t) \right) ={\widehat{p}}_{H}\left( r\left( t\right) ;\,A\left( t\right) ,M\left( t\right) ,B\left( t\right) \right) =\frac{(r+\delta )^{\beta }w^{1-\beta }}{\beta ^{\beta }(1-\beta )^{1-\beta }}. \end{aligned}$$

Reinserting this, we obtain the equilibrium utilisation of health care, as

$H^{d}\left( p_{H}^{*}\left( t\right) ;\,M\left( t\right) ,B\left( t\right) \right) ={\widehat{H}}\left( r(t);\,A\left( t\right) ,M\left( t\right) ,B\left( t\right) \right)$. Using (44) we now can determine $L_{H}^{*}\left( t\right) ={\widehat{L}}_{H}\left( p_{H}^{*}\left( t\right) ,w^{*}\left( t\right) ,H_{d}^{*}(t)\right) ={\widehat{L}} _{H}\left( r(t);\,A\left( t\right) ,M\left( t\right) ,B\left( t\right) \right)$. The labour market equilibrium then determines

$$\begin{aligned} L_{Y}^{*}\left( t\right) =L\left( t\right) -L_{H}^{*}\left( t\right) , \end{aligned}$$

where $L\left( t\right) ={\widehat{L}}\left( r(t);\,A\left( t\right) ,M\left( t\right) ,B\left( t\right) \right)$.^43^ This implies the restriction

$$\begin{aligned} {\widehat{L}}\left( r(t);\,A\left( t\right) ,M\left( t\right) ,B\left( t\right) \right) \ge {\widehat{L}}_{H}\left( r(t);\,A\left( t\right) ,M\left( t\right) ,B\left( t\right) \right) . \end{aligned}$$

Given this is satisfied, we now have all inputs and outputs as functions of $r\left( t\right)$ and the states $\left\{ A\left( t\right) ,M\left( t\right) ,B\left( t\right) \right\}$.

## Appendix 4: Impact of Medical Technology

*Impact on the demand for health care and on the VOL:* Totally differentiating the first-order condition $-\phi (a,t)p_{H}\left( t\right) -\mu _{h}\left( a,t\right) \psi \left( a,t\right) =0$ with respect to the state of technology $M\left( t\right)$ gives

$$\begin{aligned} -\phi dp_{H}-\left( \mu _{hh}dh+\mu _{hM}dM\right) \psi -\mu _{h}d\psi =0 \end{aligned}$$

which transforms to

49 $$\begin{aligned} \frac{dh\left( a,t\right) }{dM\left( t\right) } &= \frac{-1}{\mu _{hh}}\left[ \mu _{hM}+\frac{1}{\psi \left( a,t\right) }\left( \phi \frac{dp_{H}\left( t\right) }{dM\left( t\right) }+\mu _{h}\left( a,t\right) \frac{d\psi \left( a,t\right) }{dM\left( t\right) }\right) \right] \nonumber \\ &= \frac{-1}{\mu _{hh}}\left[ \mu _{hM}+\mu _{h}(a,t)\left( \frac{1}{\psi (a,t)}\frac{d\psi (a,t)}{dM(t)}-\frac{1}{p_{H}(t)}\frac{dp_{H}(t)}{dM\left( t\right) }\right) \right] . \end{aligned}$$

The impact of technology on the private value of life, as defined in (14), is given by

50 $$\begin{aligned}&\frac{d\psi \left( a,t\right) }{dM\left( t\right) }=\int _{a}^{\omega } \frac{dv\left( {\widehat{a}},t+{\widehat{a}}-a\right) }{dM\left( t\right) } R\left( {\widehat{a}},a\right) +v\left( {\widehat{a}},t+{\widehat{a}}-a\right) \frac{dR({\widehat{a}},a)}{dM}d{\widehat{a}} \nonumber \\&\quad =\int _{a}^{\omega }\frac{dv\left( {\widehat{a}},t+{\widehat{a}}-a\right) }{ dM\left( t\right) }R\left( {\widehat{a}},a\right) \nonumber \\&\qquad -v\left( {\widehat{a}},t+ {\widehat{a}}-a\right) R({\widehat{a}},a)\int _{a}^{{\widehat{a}}}\frac{dr(t+\hat{ {\hat{a}}}-a)}{dM}d\hat{{\hat{a}}}d{\widehat{a}} \end{aligned}$$

where

$$\begin{aligned} \frac{dv\left( a,t\right) }{dM\left( t\right) } &= \left( \frac{ u_{c}u_{c}-uu_{cc}}{u_{c}^{2}}\right) \frac{dc\left( a,t\right) }{dM\left( t\right) }=\left( 1-\frac{uu_{cc}}{u_{c}^{2}}\right) \frac{dc\left( a,t\right) }{ dM\left( t\right) }. \end{aligned}$$

Note, that $( 1-\frac{uu_{cc}}{u_{c}^{2}})$ is always positive: Assuming *b* is sufficiently large and $c>c_0$, $u(c)=b+\frac{(c-c_0)^{1-\sigma }}{1-\sigma } > 0$, $u_c = (c-c_0)^{-\sigma } > 0$ and $u_{cc}= -\sigma (c-c_0)^{-\sigma -1} < 0$. Equation (25) is then obtained by inserting (50) into (49).

*Impact on the wage rate and price for health care:*^44^ In the following we derive Eq. (31) and (32). We use Eq. (47) from “Appendix 3” and obtain

$$\begin{aligned} \frac{dw}{dM} &= -A\alpha ^{\frac{1}{(1-\alpha )}}(r+\delta )^{\frac{1}{ (\alpha -1)}}\frac{dr}{dM}=-A\left( \frac{\alpha }{r+\delta }\right) ^{\frac{1}{(1-\alpha )}}\frac{dr }{dM}=-\frac{\alpha }{1-\alpha }\frac{w}{r+\delta }\frac{dr}{dM}. \end{aligned}$$

Hence, given Eq. (48), it then holds, that

$$\begin{aligned} \frac{dp_{H}}{dM} &= \frac{1}{\beta ^{\beta }(1-\beta )^{1-\beta )}}\left[ \beta (r+\delta )^{\beta -1}\frac{dr}{dM}w^{1-\beta }+(r+\delta )^{\beta }(1-\beta )w^{-\beta }\frac{dw}{dM}\right] \\ &= \frac{1}{\beta ^{\beta }(1-\beta )^{1-\beta )}}\frac{dr}{dM}(r+\delta )^{\beta -1}w^{1-\beta }\left[ \beta -(1-\beta )\frac{\alpha }{1-\alpha } \right] \\ &= \frac{p_{H}}{r+\delta }\frac{\beta -\alpha }{1-\alpha }\frac{dr}{dM}. \end{aligned}$$

*Impact on the GDP per worker:* GDP is defined as the sum of output value in the health care sector, $p_{h}F$, and in the final good sector, *Y*. Hence, *GDP* per unit of labour is given by

$$\begin{aligned} \frac{GDP}{L}=\frac{1}{L}\Big (p_{H}F+Y\Big )=\frac{Y}{L}\left( \frac{p_{H}F}{Y }+1\right) . \end{aligned}$$

Defining the employment share of the final goods sector as $\lambda :=\frac{ L_{Y}}{L}$ one can then show that

51 $$\begin{aligned} \frac{GDP}{L}=\left[ \frac{1-\alpha }{1-\beta }\frac{1-\lambda }{\lambda }+1 \right] A^{1-\alpha }\left( \frac{K_{Y}/L_{Y}}{K/L}\right) ^{\alpha }\lambda \left( \frac{K}{L}\right) ^{\alpha } \end{aligned}$$

where we used Eq. (29) together with

52 $$\begin{aligned} \frac{p_{H}F}{Y}=\frac{1-\alpha }{1-\beta }\frac{1-\lambda }{\lambda } \end{aligned}$$

which follows from dividing Eq. (44) by (42) and rearranging. The economy-wide capital-intensity can be expressed as

53 $$\begin{aligned} \frac{K}{L} &= \frac{K_{Y}+K_{H}}{L_{Y}+L_{H}}=\frac{\alpha Y+\beta p_{H}F}{ (1-\alpha )Y+(1-\beta )p_{H}F}\frac{w}{r+\delta }\nonumber \\ &= \frac{(1-\alpha )\beta +(\alpha -\beta )\lambda }{\left( 1-\alpha \right) \left( 1-\beta \right) } \frac{w}{r+\delta } \end{aligned}$$

where Eq. (52) is employed. Using, in addition, (45) we can write

$$\begin{aligned} \frac{K_{Y}/L_{Y}}{K/L}=\frac{\alpha \left( 1-\beta \right) }{(1-\alpha )\beta +(\alpha -\beta )\lambda }. \end{aligned}$$

Substituting this into (51) and rearranging we obtain

$$\begin{aligned} \frac{GDP}{L}=\frac{1-\alpha +\left( \alpha -\beta \right) \lambda }{1-\beta }A^{1-\alpha }\left[ \frac{\alpha \left( 1-\beta \right) }{\beta \left( 1-\alpha \right) +\left( \alpha -\beta \right) \lambda }\right] ^{\alpha }\left( \frac{K}{L}\right) ^{\alpha } \end{aligned}$$

as reported in Eq. (33). Taking the total derivative with respect to medical technology then yields

$$\begin{aligned} \frac{d}{dM}\left( \frac{GDP}{L}\right) &= (\alpha -\beta )\frac{GDP}{L} \left[ \frac{1}{1-\alpha +\left( \alpha -\beta \right) \lambda }-\frac{ \alpha }{\beta \left( 1-\alpha \right) +\left( \alpha -\beta \right) \lambda }\right] \frac{d\lambda }{dM} \\&\quad +\alpha \frac{GDP/L}{K/L}\frac{d}{dM}\left( \frac{K}{L}\right) \\ &= \frac{-\left( 1-\alpha \right) \left( \alpha -\beta \right) ^{2}\left( 1-\lambda \right) }{\left[ 1-\alpha +\left( \alpha -\beta \right) \lambda \right] \left[ \beta \left( 1-\alpha \right) +\left( \alpha -\beta \right) \lambda \right] }\frac{GDP}{L}\frac{d\lambda }{dM} \\&\quad +\alpha \frac{GDP}{K}\frac{d}{dM}\left( \frac{K}{L}\right) , \end{aligned}$$

as reported in in Eq. (34) in the main body of the paper. Note, that the denominator $\left[ 1-\alpha +\left( \alpha -\beta \right) \lambda \right] \left[ \beta \left( 1-\alpha \right) +\left( \alpha -\beta \right) \lambda \right]$ is positive, as follows from Eq. (53).

## Appendix 5: Solving the Numerical Problem

We pursue the following steps towards tracing out the numerical solution, sketched here for the benchmark scenario:

1. We derive from the first-order condition for consumption (12) the relationship
  54 $$\begin{aligned}&\left[ c\left( a,t_{0}+a\right) -c_{0}\right] ^{-\sigma }\nonumber \\&\quad =\left[ c\left( 0,t_{0}\right) -c_{0}\right] ^{-\sigma }\exp \left\{ \int _{0}^{a}\left[ \rho -r(t_{0}+{\hat{a}})+\mu ({\hat{a}})\right] d{\hat{a}}\right\} . \end{aligned}$$
2. We derive the life-cycle budget constraint
  $$\begin{aligned} \int _{0}^{\omega }\left[ \begin{array}{c} w\left( t_{0}+a\right) l\left( a\right) -c\left( a,t_{0}+a\right) +\pi (a,t) \\ -\phi (a,t)p_{H}\left( t_{0}+a\right) h\left( a,t_{0}+a\right) -\tau (a,t)+s(t_{0}+a) \end{array} \right] R\left( a,0\right) da=0, \end{aligned}$$
  with $R\left( a,0\right)$ as given by (16). We then insert (54) and obtain the consumption level
  55 $$\begin{aligned}&c\left( 0,t_{0}\right) -c_{0}\nonumber \\&\quad =\frac{\int _{0}^{\omega }\left[ \begin{array}{c} w\left( t_{0}+a\right) l\left( a\right) -c_{0}+\pi (a,t) \\ -\phi (a,t)p_{H}\left( t_{0}+a\right) h\left( a,t_{0}+a\right) -\tau (a,t)+s(t_{0}+a) \end{array} \right] R\left( a,0\right) da}{\int _{0}^{\omega }\exp \left\{ \int _{0}^{a} \left[ \frac{1-\sigma }{\sigma }r(t_{0}+{\hat{a}})-\frac{\rho +\mu ({\hat{a}})}{ \sigma }\right] d{\hat{a}}\right\} da} \nonumber \\ \end{aligned}$$
  for an individual born at $t_{0},$ contingent on the stream of health care, $h\left( a,t_{0}+a\right) ,$ and the set of prices $\left\{ w\left( t_{0}+a\right) ,r(t_{0}+a),p_{H}\left( t_{0}+a\right) \right\}$ over the interval $\left[ t_{0},t_{0}+\omega \right] .$
3. We derive from the first-order condition for health care (13 ) a vector of age-specific demand levels
  56 $$\begin{aligned}&h(a,t_{0}+a)\nonumber \\&\quad =\left( \frac{\lambda _{s}(a,t_{0}+a)\left[ c(a,t_{0}+a)-c_{0} \right] ^{\sigma }{\tilde{\mu }}(a)\eta (a)\epsilon (a)M(t_{0}+a)^{\epsilon (a)} }{\phi (a,t)p_{H}(t_{0}+a)}\right) ^{\frac{1}{1-\epsilon (a)}} \end{aligned}$$
  for all $$a\in \left[ 0,\omega \right] .$$
4. We show in “Appendix 3” that the set of prices $$\left\{ w\left( t_{0}+a\right) ,p_{H}\left( t_{0}+a\right) \right\} $$ as well as all input and output quantities can be expressed in terms of the interest rate $$ r(t_{0}+a)$$ alone.
5. Using (54) together with (56) we can calculate the life-cycle allocation for consumption, $$c\left( a,t_{0}+a\right) $$, depending on the allocation for health expenditures, $$ h(a,t_{0}+a)$$, $$\forall a\in \left[ 0,\omega \right] $$ and on the set of prices $$\left\{ w\left( t_{0}+a\right) ,r(t_{0}+a),p_{H}\left( t_{0}+a\right) \right\} $$ over the interval $$\left[ t_{0},t_{0}+\omega \right] $$. Vice versa, the allocation of health expenditures can be calculated from the allocation of consumption and the macroeconomic prices.
6. We apply these calculations on initial guesses of *c* and *h* iteratively. We then use the results as an initial guess to the age-structured optimal control algorithm, as presented in Veliov (2003). This yields an optimal allocation of individual consumption and health expenditures contingent on an initially assumed $r(t_{0}+a)$.
7. Drawing on this, we apply the following recursive approximation algorithm: (1) Guess an initial interest rate $r(t_{0}+a)$ and derive the optimal life-cycle allocation. (2) Based on this, calculate the market interest rate $r^{*}(t_{0}+a)$ from the capital market equilibrium $K^{d}\left( r(t_{0}+a),{\widehat{w}}\left( r(t_{0}+a)\right) \right) =K^{s}\left( r(t_{0}+a)\right) .$ (3) Adjust the initial interest rate, so that it approaches $r^{*}(t_{0}+a)$, e.g. by setting $r_{1}(t_{0}+a):=r_{0}(t_{0}+a)+\epsilon (r^{*}(t_{0}+a)-r_{0}(t_{0}+a)),\quad \epsilon \in (0,1]$. The process converges to an interest rate for which households optimise and capital demand equals capital supply. The output market clearing condition, $Y(t_{0}+a)=C(t_{0}+a)+ {\dot{K}}(t_{0}+a)+\delta K(t_{0}+a)$ then determines the dynamics of the capital stock to the next period. (4) This process is reiterated in a recursive way, employing a solution algorithm based on Newton’s method. Equations (54)–(56) allow us to verify ex-post an optimum life-cycle allocation for the focal cohort born at $t_{0}$. While the numerical algorithm cannot determine in a precise way the optimal allocation for other cohorts, it nevertheless structures the allocation in a way that approximates the optimum for all cohorts.

We solve the model over a time horizon of 300 periods. The number of periods is chosen to be this large in order for the initial and final conditions of the model simulation not to matter for the medical innovation occurring at $t=150$. This implies, that the economy is in steady state well before the shock occurs, and that the transition to the new steady state is fully achieved before the end of the simulated time horizon.

## Footnotes

1 Other important drivers include income (Hall and Jones 2007) and social security (Zhao 2014).

2 The analysis also reveals an important complementarity between medical progress and income, which explains 57% of the increase in spending.

3 According to an earlier finding by Suen (2009) the compound effect of medical progress and income growth explains all of the expenditure increase 1950–2001.

4
Skinner et al. (2006) and Chandra and Skinner (2012) take a more nuanced view, showing that whether or not welfare gains arise from the adoption of new medical technologies depends both on the nature of technology as well as on the organisation of the health care system into which it is adopted.

5 Indeed, Faere et al. (1997) and Spitalnic et al. (2016) identify very low productivity growth in the health care sector, suggesting that medical progress is predominantly due to product innovation rather than process innovation. When surveying the role of medical progress for health care expenditure growth, Chandra and Skinner (2012) come to a similar conclusion of medical innovations being strongly biased to being product rather than process innovations.

6 This is consistent with empirical evidence provided by Bloom et al. (2007) and De Nardi et al. (2010).

7 The Bureau of Economic Analysis (BEA) reports that over the time span 1980–2000 medical prices have risen 1.3-times faster than the consumption price index.

8 In particular, models that assume infinitely-lived agents are abstracting altogether from a saving response to health-induced changes in longevity. As e.g. Bloom et al. (2007) and De Nardi et al. (2010) show, however, such a response is empirically relevant.

9 OLG models with realistic demography have been applied other contexts (see e.g. Boucekkine et al. 2002; D’Albis 2007; Heijdra and Romp 2008, 2009a, b; Heijdra and Mierau 2012; Mierau and Turnovsky 2014). These models do not involve endogenous health and survival.

10 Similar to Frankovic and Kuhn (2018) and Boehm et al. (2018) also study R&D driven medical progress. Jones (2016) studies the interaction of conventional and life-saving R&D but does so within a social planner context.

11
Zweifel et al. (2005) provide empirical evidence of decreasing returns to health expenditure in the reduction of mortality. The decreasing returns assumption is also reflected in other empirical work on the relationship between health care and mortality (e.g. Lichtenberg 2004; Hall and Jones 2007).

12 Here, we define $\overset{\cdot }{X}(a,t):=\frac{dX(a+s,t+s)}{ds}$ to be the derivative of the state $X=k,S$ with respect to both age and time along the 45-degree line in the Lexis-diagram.

13 It might be argued that health status should show up in the mortality function. The key difference this would make in terms of the mechanics of the model is that through its impact on health status the stream of past health care $h({\hat{a}})$ with ${\hat{a}}$ in [0; *a*) would also have an impact on mortality at age *a* besides the level of current (acute) health care *h*(*a*). This would add a motive for the individual to invest in preventive health care. Given that preventive care, even when defined broadly, accounts only for around 9% of health care expenditure (Miller et al. 2008), we believe these effects to be of second order and, therefore, omit them for the sake of a leaner analysis.

14 In a slight abuse of notation, *N*(*t*) denotes the population size at time *t* , whereas *N*(*a*, *t*) represents the size of the cohort aged *a* at time *t*.

15 In order to ease on notation, we will subsequently refer to the shortcut $\mu (a,t)$ for $\mu (a,h(a,t),M(t)).$

16 In order to ease on notation, we will subsequently refer to the shortcuts *u*(*a*, *t*) for $u(c(a,t)-c_{0})$ and $u_{c}(a,t)$ for $u_{c}(c(a,t)-c_{0}).$

17 Here and in the following, ${\widehat{a}}$ refers to an age that is distinct from age *a*, whereas $\widehat{{\widehat{a}}}$ is employed to count age/time within an integral.

18 The VOL as we calculate it here differs from the typical representation of the value of a statistical life as e.g. in Shepard and Zeckhauser (1984), Rosen (1988), Johansson (2002), or Murphy and Topel (2006) in as far as (1) the discount factor does not include the mortality rate; and (2) the VOL does not include the current change to the individual’s wealth, $lw-c-h-\tau +\pi +s.$ Both of these features are due to the absence of an annuity market.

19 It is easy to verify that a decline in the employment share $\lambda \left( t\right)$ will in optimum be accompanied by a decline in $K_{Y}\left( t\right) /K\left( t\right) .$

20 See Frankovic et al. (2017) for the analysis of a second experiment where the medical innovation is anticipated.

21 We follow the bulk of the literature and neglect life-cycle decisions during childhood.

22 For the stable population within a steady state the rate $\nu$ should equal the rate of population growth. According to the World Bank Development Indicators, the latter stood at 1.11% in the year 2000, making this a good match.

23 Note that *I*(*a*) only influences mortality (because $\mu _{h}$ is independent of *I*), whereas $\eta (a)$ also influences the demand for health care. The 2003 mortality rates are again taken from HMD. Due to limited data availability, we use health expenditure data for the year 2000, as provided in Meara et al. (2004).

24 Dollar values are to be interpreted as year 2003 Dollars throughout.

25 We introduce the minimum consumption to improve the fit of the consumption profile. While the minimum level is never hit in optimum, it helps to avoid an unrealistically sharp drop in consumption and consequently debt repayment during the oldest ages. The level of the minimum consumption profile is set such that assets of the elderly never fall below zero.

26 This value was calculated based on the following data of the US economy in 2003: Share of the elderly in total health spending = 40% (NHEA); health share in the GDP = 15% (NHEA); Medicare share in the GDP = 2.3% (Zhao 2014).

27 The right-skewed hump in consumption with a relatively strong decline for ages above 70 is well in line with the profile for private consumption (net of health care and education expenditures) calculated from the US National Transfer Accounts for the year 2003 (see Tung 2011; Figure 6.13).

28 Also note that a hump-shaped pattern is not inconsistent with the finding that health care utilisation/expenditure increases with the closeness to death (e.g. Zweifel et al. 1999). This is because the “cost of dying” itself is declining with age for the highest ages (e.g. Cutler 2007).

29 A close glance reveals a very small amount of debt during young ages, which, strictly speaking, is not fully consistent with the absence of an annuity market. However, we consider the level of debt to be negligible for all practical purposes. This is both for its magnitude and for the fact that it is incurred for an age interval with negligible mortality.

30 The health share is calculated as $\frac{p_{H}(t)H(t)}{GDP(t)}=\frac{p_{H}(t)H(t)}{p_{H}(t)H(t)+Y(t)}$.

31 See Frankovic and Kuhn (2018, 2019) for a calibration of the dynamics of the US economy.

32 We allow the first 100 periods for the economy to reach the initial benchmark steady-state.

33 To see this note that

$$\begin{aligned} \mu _{h}(a,t) &= -{\widetilde{\mu }}(a)\eta (a)\epsilon (a)M(t)^{\epsilon (a)}h(a,t)^{\epsilon (a)-1}<0, \\ \mu _{M}(a,t) &= -{\widetilde{\mu }}(a)\eta (a)\epsilon (a)M(t)^{\epsilon (a)-1}h(a,t)^{\epsilon (a)}<0, \\ \mu _{hM}(a,t) &= -{\widetilde{\mu }}(a)\eta (a)(\epsilon (a))^{2}[M(t)h(a,t)]^{\epsilon (a)-1}<0. \end{aligned}$$

34
Roham et al. (2014) also show that the bulk of the expenditure increase associated with more intensive treatments lies with the age groups 55 and over with a peak increase within the age group 75–79 (see their Figure 6). Qualitatively, this is very similar to the age-profile of the expenditure increase in our model.

35 A partial equilibrium perturbation of $p_{H}$ enables us to determine the price elasticity of per-capita health care expenditures for the benchmark calibration. We find a price elasticity of $-0.3$, which is close to the estimated mean elasticity of − 0.2 determined in the RAND Health Insurance Experiment (Manning et al. 1987).

36
Fonseca et al. (2013) find within a partial equilibrium model calibrated to the US context that an increase of health care expenditure by 247% and an increase in life expectancy by 9.6 years over the time span 1965–2005 could be attributed to medical change. Assuming linearity, this would imply that an innovation-induced increase in life expectancy by 1.1 years would be associated with an increase in expenditure by 28%, which is consistent with our partial equilibrium result.

37
Hult et al. (2018) provide a detailed study of the price patterns for innovative health care treatments. They find that while innovative treatments tend to have a higher quality-adjusted price than incumbent treatments at the point of their market entry, a reduction of the price in excess of 4% by the time that the innovative technology becomes incumbent is sufficient for the price of successive incumbent treatments to decline over time. While our macro approach is lacking the detailed modelling of the price wedge between innovative and incumbent treatments, it is fully consistent with the price dynamics that arise for quality-improvements in a sequence of incumbent products.

38 The increase in the price of health care is well in line with the fact that the US consumer price index (CPI) for medical care consistently grows in excess of the CPI for all items (see US Bureau of Labor Statistics).

39 The medical innovation raises the remaining life expectancy at age 20 by 1.0 years from 58.04 years (and, thus, by 1.3%) and remaining life expectancy at age 65 by 0.81 years from 18.02 years (and, thus, by 4.5%).

40 Note, however, that within a decentralised economy with R&D-driven growth a la Romer (1990) the increase in the capital intensity of final goods production that follows the absorption of (relatively more) labour by a growing health care sector provides a stimulus for conventional R&D (Kuhn and Prettner 2016).

## References

[CR1] Abeliansky AL, Strulik H (2018). How we fall apart: Similarities of human aging in 10 European countries. Demography.

[CR2] Acemoglu D, Guerrieri V (2008). Capital deepening and nonbalanced economic growth. Journal of Political Economy.

[CR3] Aldy JE, Viscusi WK (2008). Adjusting the value of a statistical life for age and cohort effects. Review of Economics and Statistics.

[CR4] Baker L, Birnbaum H, Geppert J, Mishol D, Moyneur E (2003). The relationship between technology availability and health care spending. Health Affairs.

[CR5] Baumol WJ (1967). Macroeconomics of unbalanced growth: The anatomy of urban crisis. American Economic Review.

[CR6] Bloom DE, Canning D, Mansfield RK, Moore M (2007). Demographic change, social security systems, and savings. Journal of Monetary Economics.

[CR7] Boehm, S., Grossmann, V., & Strulik, H. (2018). *R&D-driven medical progress, health care costs, and the future of human longevity*. CESifo working paper 6897.

[CR8] Boucekkine R, de la Croix D, Licandro O (2002). Vintage human capital, demographic trends, and endogenous growth. Journal of Economic Theory.

[CR9] Bundorf MK, Royalty A, Baker LC (2009). Health care cost growth among the privately insured. Health Affairs.

[CR10] Chandra A, Skinner J (2012). Technology growth and expenditure growth in health care. Journal of Economic Literature.

[CR11] Chernew ME, Newhouse JP, Pauly MV, Barros PG (2012). Health care spending growth, chapter 15. Handbook of health economics.

[CR12] Chetty R (2006). A new method of estimating risk aversion. American Economic Review.

[CR13] Cutler DM (2004). Your money or your life: Strong medicine for America’s health care system.

[CR14] Cutler DM (2007). The lifetime costs and benefits of medical technology. Journal of Health Economics.

[CR15] Cutler DM, Huckman RS (2003). Technological development and medical productivity: The diffusion of angioplasty in New York state. Journal of Health Economics.

[CR16] Cutler DM, McClellan M, Newhouse JP, Rehmer D (1998). Are medical prices declining? Evidence from heart attack treatments. Quarterly Journal of Economics.

[CR17] D’Albis H (2007). Demographic structure and capital accumulation. Journal of Economic Theory.

[CR18] De Nardi M, French E, Jones JB (2010). Why do the elderly save? The role of medical expenses. Journal of Political Economy.

[CR19] De Nardi M, French E, Jones JB, McCauley J (2016). Medical spending by the US elderly. Fiscal Studies.

[CR20] Dunn A (2012). Drug innovations and welfare measures computed from market demand: The case of anti-cholesterol drugs. American Economic Journal: Applied Economics.

[CR21] Faere R, Grosskopf S, Lindgren B, Poullier J-P (1997). Productivity growth in health-care delivery. Medical Care.

[CR22] Fonseca, R., Michaud, P-C., Kapteyn, A., & Galama, T. (2013). *Accounting for the rise of health spending and longevity*. IZA discussion paper no. 7622. 10.1093/jeea/jvaa003 PMC7935060 33679266

[CR23] Frankovic, I., & Kuhn, M. (2018). *Health insurance, endogenous medical progress, and health expenditure growth*. TU Vienna econ working paper 01/2018. 10.1016/j.jhealeco.2022.102717 36638641

[CR24] Frankovic, I., & Kuhn, M. (2019). Access to health care, medical progress and the emergence of the longevity gap: A general equilibrium analysis. *Journal of the Economics of Ageing*, *14*, Article 100188.

[CR25] Frankovic, I., Kuhn, M., & Wrzaczek, S. (2017). *Medical progress, demand for health care, and economic performance*. TU Vienna econ working paper 08/2017.

[CR26] Hall RE, Jones CI (2007). The value of life and the rise in health spending. Quarterly Journal of Economics.

[CR27] Heijdra BJ, Mierau JO (2012). The individual life-cycle, annuity market imperfections and economic growth. Journal of Economic Dynamics and Control.

[CR28] Heijdra BJ, Romp WE (2008). A life-cycle overlapping-generations model of the small open economy. Oxford Economic Papers.

[CR29] Heijdra BJ, Romp WE (2009). Human capital formation and macroeconomic performance in an ageing small open economy. Journal of Economic Dynamics and Control.

[CR30] Heijdra BJ, Romp WE (2009). Retirement, pensions, and ageing. Journal of Public Economics.

[CR31] Hult KJ, Jaffe S, Philipson TJ (2018). How does technological change affect quality-adjusted prices in health care? Systematic evidence from thousands of Innovations. American Journal of Health Economics.

[CR32] Johansson P-O (2002). On the definition and age-dependency of the value of a statistical life. Journal of Risk and Uncertainty.

[CR33] Jones CI (2016). Life and growth. Journal of Political Economy.

[CR34] Kelly M (2017). Health capital accumulation, health insurance, and aggregate outcomes: A neoclassical approach. Journal of Macroeconomics.

[CR35] Koijen RSJ, Philipson TJ, Uhlig H (2016). Financial health economics. Econometrica.

[CR36] Kuhn M, Prettner K (2016). Growth and welfare effects of health care in knowledge based economies. Journal of Health Economics.

[CR37] Kuhn M, Wrzaczek S, Oeppen J (2010). Recognizing progeny in the value of life. Economics Letters.

[CR38] Kuhn M, Wrzaczek S, Prskawetz A, Feichtinger G (2011). Externalities in a life cycle model with endogenous survival. Journal of Mathematical Economics.

[CR39] Kuhn M, Wrzaczek S, Prskawetz A, Feichtinger G (2015). Optimal choice of health and retirement in a life-cycle model. Journal of Economic Theory.

[CR40] Lakdawalla D, Shafrin J, Lucarelli C, Nicholson S, Khan ZM, Philipson T (2015). Quality-adjusted cost of care: A meaningful way to measure growth in innovation cost versus the value of health gains. Health Affairs.

[CR41] Lichtenberg FR (2004). Sources of U.S. longevity increase, 1960–2001. Quarterly Review of Economics and Finance.

[CR42] Lucarelli, C., & Nicholson, S. (2009). *A quality-adjusted price index for colorectal cancer drugs*. NBER working paper 15174.

[CR43] Ludwig A, Schelkle T, Vogel E (2012). Demographic change, human capital and welfare. Review of Economic Dynamics.

[CR44] Manning WG, Newhouse JP, Duan N, Emmett BK, Leibowitz A, Marquis MS (1987). Health insurance and the demand for medical care: Evidence from a randomized experiment. American Economic Review.

[CR45] Martini ME, Garrett N, Lindquist T, Isham GJ (2007). The boomers are coming: A total cost of care model of the impact of population aging on health care costs in the United States by major practice category. Health Services Research.

[CR46] Meara E, White C, Cutler DM (2004). Trends in medical spending by age, 1963–2000. Health Affairs.

[CR47] Mierau JO, Turnovsky SJ (2014). Capital accumulation and the sources of demographic change. Journal of Population Economics.

[CR48] Miller G, Roehrig C, Hughes-Cromwick P, Lake C, Helmchen L, Kaestner R, Lo Sasso A (2008). Quantifying national spending on wellness and prevention. Beyond health insurance: Public policy to improve health (advances in health economics and health services research).

[CR49] Murphy KM, Topel RH (2006). The value of health and longevity. Journal of Political Economy.

[CR50] Pauly MV, Saxena A (2012). Health employment, medical spending, and long-term health reform. CES-Ifo Economics Studies.

[CR51] Reichling F, Smetters K (2015). Optimal annuitization with stochastic mortality and correlated medical costs. American Economic Review.

[CR52] Rockwood K, Mitnitski A (2007). Frailty in relation to the accumulation of deficits. Journal of Gerontology: Medical Sciences.

[CR53] Roham M, Gabrielyan AR, Archer NP, Grignon ML, Spencer BG (2014). The impact of technological intensity of service provision on physician expenditures: An exploratory investigation. Health Economics.

[CR54] Romer PM (1990). Endogenous technological change. Journal of Political Economy.

[CR55] Rosen S (1988). The value of changes in life expectancy. Journal of Risk and Uncertainty.

[CR56] Schneider, M. T., & Winkler, R. (2016). *Growth and welfare under endogenous life-time*. University of Bath: Bath Economics Research papers no. 47/16.

[CR57] Shepard DS, Zeckhauser RJ (1984). Survival versus consumption. Management Science.

[CR58] Skinner JS, Staiger DO (2015). Technology diffusion and productivity growth in health care. Review of Economics and Statistics.

[CR59] Skinner JS, Staiger DO, Fisher ES (2006). Is technological change in health care always worth it? The case of acute myocardial infarction. Health Affairs.

[CR60] Spitalnic, P., Heffler, S., Dickensheets, B., & Knight, M. (2016). *Hospital multifactor productivity: An updated presentation of two methodologies*. Office of the Actuary, Centers for Medicare & Medicaid Services, U.S. Department of Health and Human Services.

[CR61] Suen, R. M. H. (2009). *Technological advance and the growth in health care spending*. Mimeo.

[CR62] Tung A-C, Lee R, Mason A (2011). Consumption over the life-cycle: An international comparison, chapter 6. Population aging and the generational economy: A global perspective.

[CR63] Veliov VM (2003). Newton’s method for problems of optimal control of heterogeneous systems. Optimization Methods and Software.

[CR64] Viscusi WK, Aldy JE (2003). The value of a statistical life: A critical review of market estimates throughout the world. The Journal of Risk and Uncertainty.

[CR65] Warshawsky M (1988). Private annuity markets in the United States: 1919–1984. Journal of Risk and Insurance.

[CR66] Wong A, Wouterse B, Slobbe LCJ, Boshuizen HC, Polder JJ (2012). Medical innovation and age-specific trends in health care utilitzation: Findings and implications. Social Science and Medicine.

[CR67] Zhao K (2014). Social security and the rise in health spending. Journal of Monetary Economics.

[CR68] Zweifel P, Felder S, Meier M (1999). Ageing of population and health care expenditure: A red herring?. Health Economics.

[CR69] Zweifel P, Steinmann L, Eugster P (2005). The Sisyphus syndrome in health revisited. International Journal of Health Care Finance and Economics.

